# Genome sequence of Perigonia lusca single nucleopolyhedrovirus: insights into the evolution of a nucleotide metabolism enzyme in the family *Baculoviridae*

**DOI:** 10.1038/srep24612

**Published:** 2016-06-07

**Authors:** Daniel M. P. Ardisson-Araújo, Rayane Nunes Lima, Fernando L. Melo, Rollie J. Clem, Ning Huang, Sônia Nair Báo, Daniel R. Sosa-Gómez, Bergmann M. Ribeiro

**Affiliations:** 1 Laboratory of Baculovirus, Cell Biology Department, University of Brasília, Brasília, DF, Brazil; 2 Division of Biology, Kansas State University, Manhattan, KS, USA; 3 Embrapa Soja, Londrina, PR, Brazil

## Abstract

The genome of a novel group II alphabaculovirus, Perigonia lusca single nucleopolyhedrovirus (PeluSNPV), was sequenced and shown to contain 132,831 bp with 145 putative ORFs (open reading frames) of at least 50 amino acids. An interesting feature of this novel genome was the presence of a putative nucleotide metabolism enzyme-encoding gene (*pelu112*). The *pelu112* gene was predicted to encode a fusion of *thymidylate kinase* (*tmk*) and *dUTP diphosphatase* (*dut*). Phylogenetic analysis indicated that baculoviruses have independently acquired *tmk* and *dut* several times during their evolution. Two homologs of the *tmk-dut* fusion gene were separately introduced into the Autographa californica multiple nucleopolyhedrovirus (AcMNPV) genome, which lacks *tmk* and *dut*. The recombinant baculoviruses produced viral DNA, virus progeny, and some viral proteins earlier during *in vitro* infection and the yields of viral occlusion bodies were increased 2.5-fold when compared to the parental virus. Interestingly, both enzymes appear to retain their active sites, based on separate modeling using previously solved crystal structures. We suggest that the retention of these *tmk-dut* fusion genes by certain baculoviruses could be related to accelerating virus replication and to protecting the virus genome from deleterious mutation.

Large double-stranded DNA viruses exhibit high genomic plasticity and primarily evolve by both horizontal gene transfer (HGT) and gene duplication/loss^1^,^2^. In many cases, viruses take advantage of an existing cellular pathway and fully or partially incorporate it into their genome^2^. With the increasing availability of genome sequence data, HGT events have been extensively documented in several viral families. This is particularly true for members of *Baculoviridae*, a family of dsDNA viruses that mainly infect the larval stages of lepidoptera (moths and butterflies)^3^.

More than 500 different types of genes have been found in the genomes of the 70-plus baculoviruses that have been sequenced to date^4^, and many of them seem to be products of HGT events^5^,^6^. For instance, an interesting but poorly studied group of genes acquired by baculoviruses are those related to nucleotide metabolism. Some baculoviruses contain homologs of *dUTP diphosphatase* (*dut*), *ribonucleotide-diphosphate reductase* (*rnr*), and/or *thymidine monophosphate kinase* (*tmk*), but none of these genes have been characterized in baculovirus at the molecular level and there is no evidence of fitness changes associated with them. Moreover, it has been suggested that baculoviruses have independently acquired *dut* and *rnr* genes more than once during their evolution^7^.

Viruses from several groups including baculoviruses, asfarvirus, herpesviruses, poxviruses, and certain retroviruses encode dUTP diphosphatase (dUTPase) and/or thymidine monophosphate kinase (TMK) enzymes in their genome. The enzyme dUTPase is conserved in prokaryotic and eukaryotic cells and such conservation is thought to be related to the inability of DNA polymerases to discriminate between dUTP and dTTP during DNA synthesis^8^.The enzyme TMK participates in both the *de novo* and the salvage dTTP biosynthesis pathways^9^. The misincorporation of dUTP in lieu of dTTP can lead to either deleterious mutations or to futile repair cycles and DNA breakage events that kill the cell^10^. Therefore, dUTPase activity serves an essential function by hydrolyzing dUTP to dUMP and PPi, lowering the dUTP/dTTP ratio and providing substrate for the major biosynthesis pathway of dTTP^11^. Other roles for dUTPases have been demonstrated including transposase-like activity, regulation of the immune system, autoimmunity, and apoptosis, suggesting that they also perform regulatory functions^12^.

In 1988, larvae of *P. lusca* showing symptoms of baculovirus infection were collected in Argentina and analyzed for the presence of a baculovirus^13^. The viral etiology was confirmed by light and electron microscopy which revealed large numbers of polyhedra-like particles with singly-enveloped occlusion-derived viruses^13^. This virus was then used for the control of this insect pest in 2,362 ha (1993) in the province of Corrientes, Argentina^13^. So far, *P. lusca* is not of great agricultural interest, but it does cause occasional damage on crops of Paraguay tea (*Ilex paraguariensis*) and Krug’s holly (*I. krugiana*), genipapo (*Genipa americana*), and coffee (*Coffea arabica*) in Brazil^14^, Argentina, Puerto Rico, Cuba, and USA (The Natural History Museum, http://www.nhm.ac.uk). In this work, we sequenced the complete genome of PeluSNPV and established its phylogeny to other baculoviruses. Furthermore, a *tmk*-*dut* fused gene was found in the PeluSNPV genome which led us to reconstruct the phylogeny of *dut* and *tmk* genes in the *Baculoviridae* family. When either the PeluSNPV *tmk-dut* fusion gene or another baculovirus *tmk-dut* homolog were inserted into the baculovirus Autographa californica multiple nucleopolyhedrovirus (AcMNPV), which naturally lacks nucleotide metabolism genes, accelerated virus progeny production, virus genome replication, and viral gene expression were observed. These results lead us to hypothesize that the reason why nucleotide metabolism genes, especially *tmk*-*dut*, are fixed in some baculovirus genomes may be due to their involvement in synchronizing the cell cycle state, controlling the dUTP/dTTP ratio, or altering the expression or function of cellular nucleotide metabolism enzymes, thereby enabling faster virus replication and protecting the virus progeny from deleterious mutation.

## Results

### Structural analysis

For structural analysis, we performed a scanning electron microscopy (SEM) of purified occlusion bodies (OBs) of PeluSNPV. Mature OBs of irregular shape and size were observed (Fig. S1A). Immature OBs revealed rod-shaped indentations on the surfaces of OBs that likely corresponded to ODV that were lost during isolation (inset, Fig. S1A). Furthermore, restriction analysis of the viral DNA revealed that PeluSNPV was probably a novel virus, since no similar restriction profile was found when compared *in silico* with other sequenced baculoviruses (Fig. S1B).

### Genome features and phylogeny of PeluSNPV

**Figure 1:**
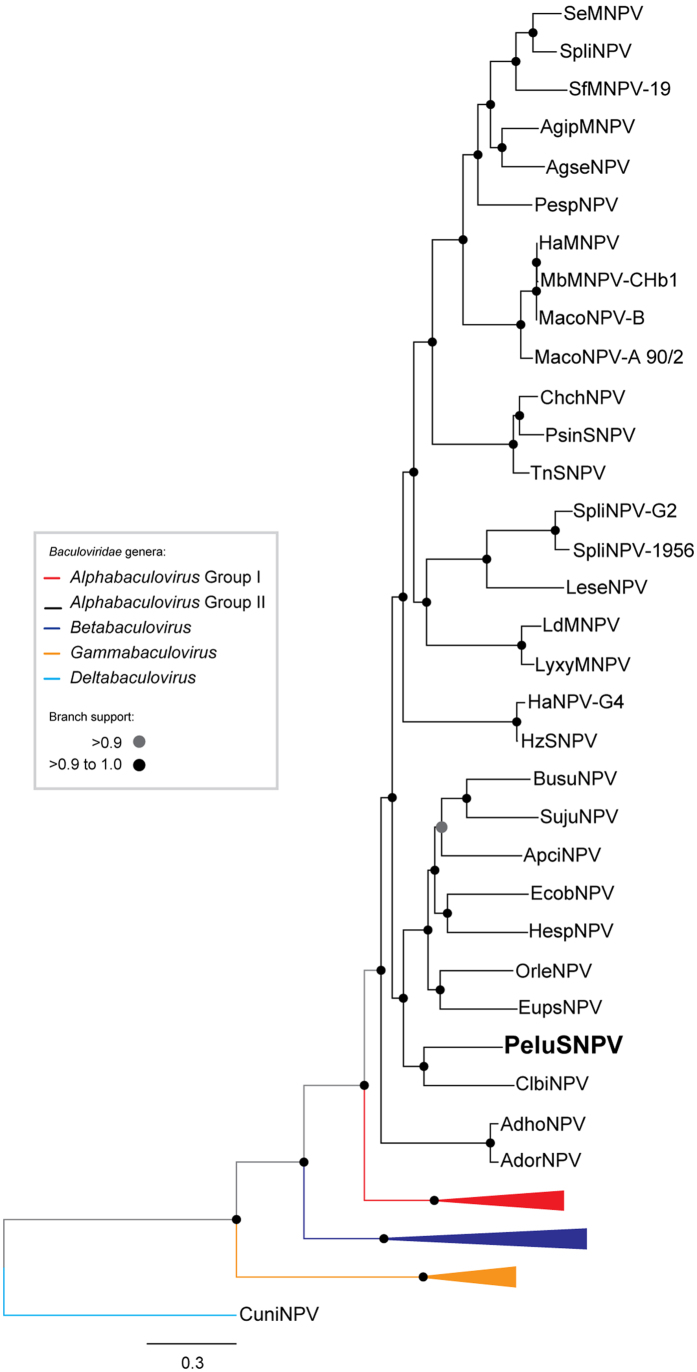
PeluSNPV is a Group II alphabaculovirus. Maximum likelihood inference based on the concatenated amino acid sequences of 37 core proteins of all complete baculovirus genomes (Table S2). The branch support was determined by a SH-like method. Some branches were collapsed for clarity: *Gammabaculovirus* (orange), *Betabaculovirus* (dark blue), and group I *Alphabaculovirus* (red). The deltabaculovirus CuniNPV was used as the root (light blue). PeluSNPV (boldface) belongs to the genus *Alphabaculovirus* and clustered with another sphingid-infecting group II alphabaculovirus, ClbiNPV.

**Figure 2:**
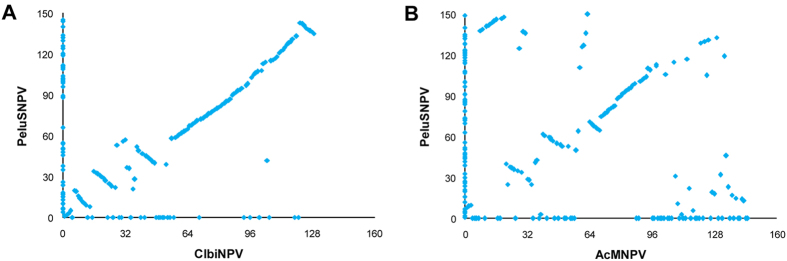
Gene content and synteny of PeluSNPV compared to other two species. (**A**) PeluSNPV was compared to ClbiNPV, another sphingid-infecting baculovirus. (**B**) PeluSNPV was compared to the baculovirus type species, AcMNPV.

The entire genome of PeluSNPV was sequenced using 454 technology (Genbank accession number KM596836) and over 18,807 single-end reads were obtained. After size and quality trimming, 18,355 reads (mean size of 356.6 ± 147.1 bp) were used for *de novo* assembly with a pairwise identity of 96.3%. The genome mean coverage was 50.4 ± 12.5 bases/site. The PeluSNPV genome was shown to contain 132,831 bp with a G+C content of 39.6%. We annotated 145 putative ORFs encoding polypeptides of at least 50 amino acid residues that started with a Met (Table S1). Eighteen of these were shown to be unique in baculoviruses and had no predicted amino acid motifs (*pelu004*, *pelu006*, *pelu010*, *pelu017*, *pelu018*, *pelu026*, *pelu035*, *pelu048*, *pelu054*, *pelu055*, *pelu089*, *pelu099*, *pelu100*, *pelu101*, *pelu119*, *pelu120*, *pelu140*, and *pelu144*). All of the currently defined 37 baculovirus core genes were found and, based on phylogenetic analysis using the concatenated alignment of the core genes from the completely sequenced baculoviruses (Table S2), PeluSNPV was found to belong to the genus *Alphabaculovirus* and to cluster most closely with Clanis bilineata nucleopolyhedrovirus (ClbiNPV) (Fig. 1). The average percent nucleotide identity of the 37 PeluSNPV core genes with ClbiNPV was 58%. The branch length separating this virus from its closest relatives is in a range that is comparable to the branch lengths separating viruses in other recognized alphabaculovirus species. Furthermore, when the gene content of PeluSNPV was compared to both ClbiNPV (Fig. 2A) and AcMNPV (Fig. 2B) by gene parity plot, many inversions, deletions, and insertions were observed relative to the genomes of these related species. The gene order was not strictly conserved between PeluSNPV and ClbiNPV, and four major inversions were detected (Fig. 2A). Although these viruses are closely related to each other, each contains several unique genes. The *lef-8*, *lef-9*, and *polh* pairwise distances of the viral sequences of PeluSNPV to other alphabaculoviruses for both single locus and concatenated alignment are well in excess of 0.05 substitutions/site, fulfilling the criteria for a novel baculovirus species^15^.

### Tandem repeat elements

In the PeluSNPV genome we found two homologous regions (*hr1* and *hr2*) and one direct repeat (*dr*) as tandem repeat elements. The *hrs* of baculoviruses are usually associated with DNA replication, homologous recombination, and gene transcription enhancement^5^,^16^,^17^. In the PeluSNPV genome, the *hrs* were located in the genome in an opposite orientation to each other with the *hr1* (738 bp) being about twice as long as *hr2* (370 bp). Both *hrs* presented one common imperfect repeat unit of about 100 bp, which was repeated four times in *hr1* and twice in *hr2*. By alignment, the overall pairwise nucleotide identity of this repeat unit was 90.3%. Moreover, regarding *hr1*, we found that the four repeat units associated themselves with four other repeats of 174, 211, 211, and 142 bp which were not found in *hr2*. On the other hand, we found a *dr* that was 481 bp in length with a perfect repeat unit of 12 nt repeated 24 times. The *dr* found in the genome of PeluSNPV is a non-typical homologous region not found in any other baculovirus genome, to our knowledge. ClbiNPV, the closest relative of PeluSNPV, presented only non-typical *hrs* in its genome, as observed for some alphabaculoviruses^18^,^19^,^20^. Interestingly, the first inversion observed in the PeluSNPV genome was located between two tandem repeat elements, the *dr* and *hr1*.

### Gene content

Baculovirus genes can be divided into different categories according to their function including genes related to replication and transcription, structural genes and auxiliary genes^5^. By BLASTX analyses, we found that the PeluSNPV genome contained homologs of AcMNPV DNA replication factors, transcription factors and structural genes associated with both BV and ODV phenotypes (Table S3). Importantly, the genus *Alphabaculovirus* is divided into group I and II, originally based on the alignment and phylogeny of the *polyhedrin*^21^ gene sequence and later confirmed through other gene sequence phylogenies^3^. The BVs of alphabaculoviruses contain two major fusion proteins in their envelopes, GP64 for viruses from group I and F protein for viruses from group II. PeluSNPV possesses an F protein homolog, corroborating the fact that it is an alphabaculovirus from group II.

Several known examples of auxiliary genes were observed in the PeluSNPV genome (Table S3). For instance, both *cathepsin* and *chitinase* were found in the genome in an opposite orientation, as commonly found in other baculovirus genomes. The putative chitinase presents a KTEL motif at its C-terminus, which is related to retention into the ER. The presence of these genes is consistent with the post-mortem phenotype observed for the host caterpillar infected with PeluSNPV, which includes both body melanization and liquefaction of internal tissues (data not shown). The *iap-2* (*pelu064*) and *iap-3* (*pelu102*) genes, which are usually present in the genomes of group II alphabaculoviruses and are involved in the anti-apoptotic response induced by virus infection, were also observed. However, the predicted *iap-3* (*pelu102*) homolog lacks one of the two commonly conserved Baculovirus IAP Repeat (BIR) domains in the N-terminal region (data not shown), which is involved in protein-protein interactions^22^. Furthermore, we found a homolog of a non-structural (NS) densovirus gene, *pelu104*. Homologs of this gene were previously found in three betabaculovirus genomes including Choristoneura occidentalis granulovirus (*choc025*)^23^, Cryptophlebia leucotreta granulovirus (*crle009*)^24^, and Erinnyis ello granulovirus (*erel057* and *erel100*)^25^. To our knowledge, PeluSNPV is the first alphabaculovirus harboring a densovirus-related gene. The phylogenetic reconstruction revealed that PeluSNPV probably acquired it from a betabaculovirus (data not shown). Moreover, a homolog of *he65* (RNA ligase-like gene) was also found in the PeluSNPV genome, *pelu124*. In a previous study, we reconstructed the phylogenetic history of *he65* and found that it is present in several baculovirus and two entomopoxvirus genomes. Importantly, a large and recurrent deletion observed at the C-terminal region of the putative baculovirus proteins was also observed in Pelu124^25^. Out from 29 he65 homologs from genomes of baculoviruses and entomopoxviruses, eight have the deletion. The phylogenetic analyses clustered *pelu124* with both group II alphabaculovirus and entomopoxvirus genes, while the closest baculovirus relative of PeluSNPV (*i.e.* ClbiNPV) lacks a *he65* ortholog.

### Genes related to nucleotide metabolism

Genes encoding both the large and small subunits of ribonucleotide reductase (RNR) were found in the PeluSNPV genome, *pelu145* and *pelu126*, respectively. Ribonucleotide reductase catalyzes the rate-limiting step for deoxyribonucleotide production required for DNA synthesis. The enzyme is a tetramer consisting of two large and two small subunits^26^. Several baculoviruses and other arthropod-related viruses contain these genes in their genomes including the white spot syndrome virus^27^. The *rnr* homologs often occur along with *dut* genes in baculovirus genomes^7^ but some *dut*-harboring betabaculoviruses lack the RNR enzyme (*e.g.* ErelGV)^25^.

**Figure 3:**
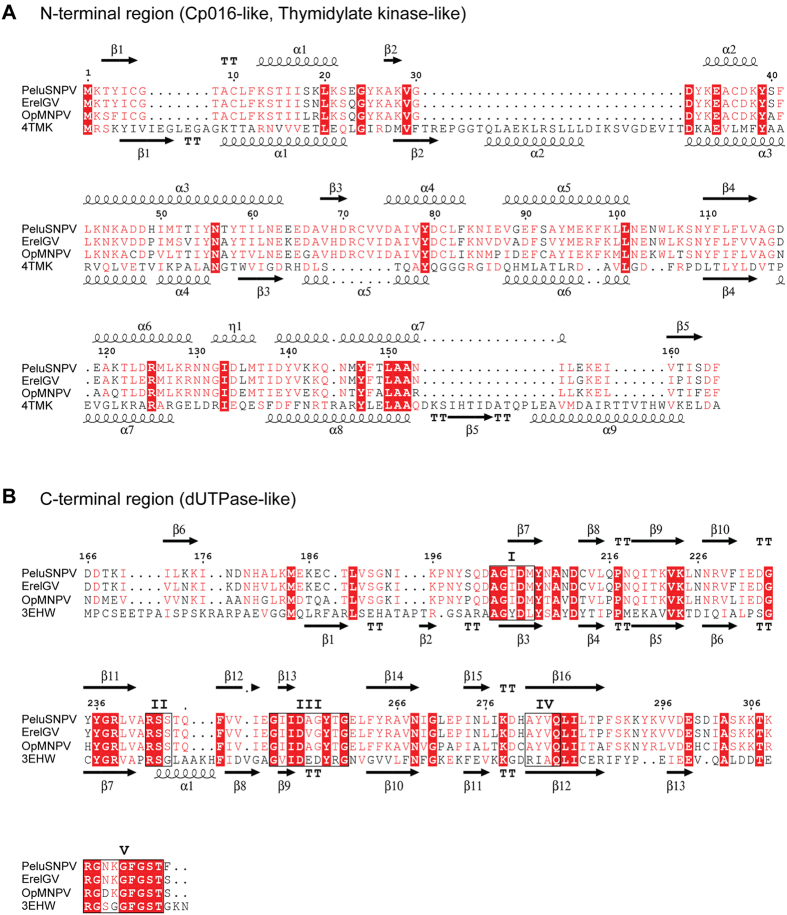
Individual structural alignments of both the TMK and dUTPase regions of TMK-dUTPase fusion proteins from PeluSNPV, ErelGV, and OpMNPV with proteins having crystal solved structures. (**A**) The predicted N-terminal region presents homology to Cp016, a putative thymidylate kinase enzyme. (**B**) Predicted C-terminal region presents homology to trimeric dUTPases. The conserved motifs are boxed in black lines from I to V. The predicted secondary structures are shown for both Pelu112 regions and the proteins with crystal solved structures. α/spirals: α-helices; β/arrows: β-sheet; tt: turns; dashed lines: no secondary structure found; red box: strictly conserved residues.

The putative ORF *pelu112* was found to be homolog of a nucleotide metabolism gene with some peculiar features. Firstly, *pelu112* was found to be a fusion of two putative genes. The predicted N-terminal region was related to the *cp016* gene of the baculovirus Cydia pomonella granulovirus (CpGV), which has homology with *thymidylate kinase* (*tmk*, Fig. 3A) whereas the predicted C-terminal region was related to *dUTP diphosphatase* (*dut*, Fig. 3B). Several secondary structures were conserved when both regions were compared to previously solved crystal structures of other TMK and dUTPase enzymes. *tmk* and *dut* homologs are present in many other baculovirus genomes as separated ORFs or, in the case of *tmk*, often fused to other genes. Secondly, *pelu112* has homologs in two other distantly related baculoviruses, ErelGV (*erel005*)^25^ and Orgyia pseudotsugata multiple nucleopolyhedrovirus (OpMNPV) (*op031*)^28^ (Fig. 3) with pairwise amino acid sequence identities of 90.2% and 74.1% respectively.

### Phylogenetic analysis of *pelu112* gene

**Figure 4:**
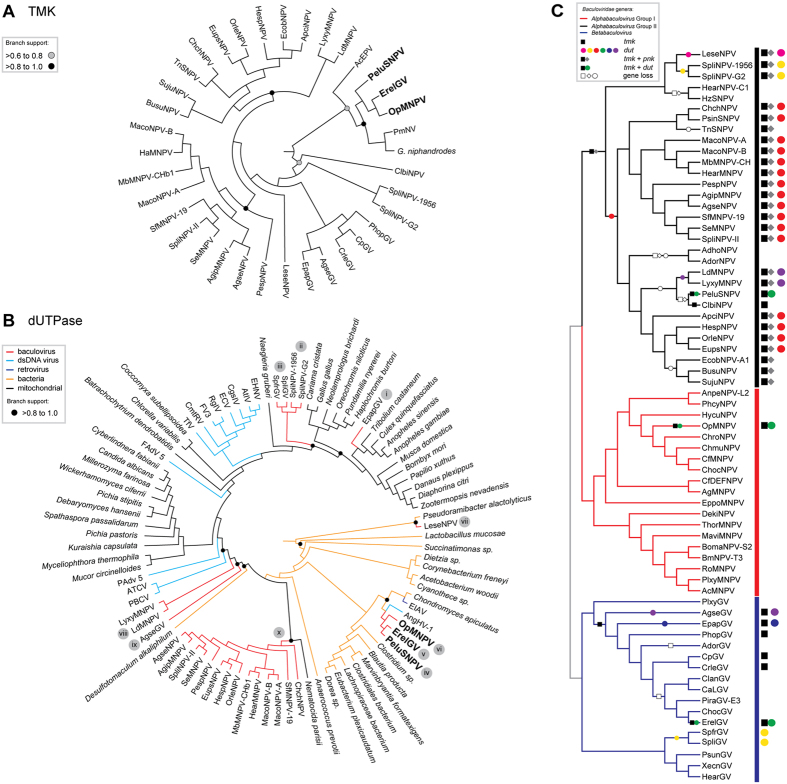
Phylogeny and evolution of the TMK and dUTPase regions of Pelu112 within the family *Baculoviridae*. (**A**) Phylogeny of cp016-like, the N-terminal portion of *tmk*-*dut*fused gene. ErelGV, OpMNPV, and PeluSNPV-derived proteins clustered together, indicating common ancestry. (**B**) Phylogeny of dUTPases in the family *Baculoviridae*. Several dUTPases clustered and seemed to be shared by several group II alphabaculoviruses. The putative independent acquisitions are numbered from *i* to *x*. (**C**) Based on the hypothetical phylogeny trees, the history of gain and loss of both *tmk* and *dut* in the family *Baculoviridae* are described in Results. For this phylogenetic analysis, we used the concatenated alignment of 37 core genes of alpha and betabaculoviruses. Filled and empty symbols represent gain and loss events, respectively. Similar events of *dut* acquisitions (circles) are shown with the same color. All the trees were midpoint rooted and presented as cladogram for clarity.

We performed separate phylogenetic reconstructions of both regions (*tmk* and *dut*) of *pelu112* (Fig. 4). In the *tmk* dataset, we included genes related to insect viruses (*e.g.* entomopoxvirus and nudivirus) and to the mealworm disease-associated apicomplexan *Gregarina niphandrodes* obtained by BLASTX. The ErelGV-, OpMNPV- and PeluSNPV-derived genes clustered together, suggesting a common ancestry (Fig. 4A). The closest relatives were both nudivirus and apicomplexan genes. Betabaculovirus-derived *tmk* genes (except ErelGV) clustered together and the same occurred with alphabaculovirus group II genes. The unique exception for alphabaculoviruses was the ClbiNPV gene, suggesting an independent HGT event.

We carried out a similar phylogenetic analysis using the predicted protein sequence of several *dut* genes from bacteria, viruses, and mitochondrial isoform genes. We found that many group II alphabaculovirus *dut* genes clustered together, forming a well-supported monophyletic clade with a fungus mitochondrial gene being the likely ancestor (Fig. 4B). Conversely, some baculovirus genes were found to be spread along the tree depicting at least nine predicted HGT events from several sources including other baculoviruses (Fig. 4B,C). The *dut* gene of Epinotia aporema granulovirus (EpapGV) seemed to be acquired from an insect mitochondrial isoform gene (*i*). The *dut* genes of Spodoptera litura granulovirus (SpliGV), Spodoptera frugiperda granulovirus (SpfrGV), Spodoptera litura nucleopolyhedrovirus AN1956 (SpliNPV-1956), and Spodoptera littoralis nucleopolyhedrovirus II (SpliNPV-II) clustered together and seem to be product of a double HGT event (*ii* and *iii*). Firstly, the gene was probably acquired from an amoeba-related mitochondrial isoform by the ancestor of either SpliGV and SpfrGV or SpliNPV-1956 and SpliNPV-II. The second event may have occurred during a co-infection scenario of a *Spodoptera sp*. host by both ancestors. Three other independent acquisitions (*iv*, *v*, and *vi*) seemed to take place in PeluSNPV, ErelGV, and OpMNPV evolution, that formed a dissimilar well-supported subclade closely related to bacteria-, lentivirus-, and adenovirus-derived *dut* genes (Fig. 4B). This acquisition probably happened once in the ancestor of one of those species (*i.e.* PeluSNPV, OpMNPV, ErelGV) and was transferred to the other baculoviruses during co-infection events. For instance, both PeluSNPV and ErelGV are sphingid-infecting baculovirus and their ancestors could potentially infect the same host. Another event appears to have occurred in Leucania separata nucleopolyhedrovirus (LeseNPV) (*vii*), with its closest relative being a bacterium. Finally, Lymantria xylina multiple nucleopolyhedrovirus (LyxyMNPV), Lymantria dispar multiple nucleopolyhedrovirus (LdMNPV) (*viii*) and Agrotis segetum granulovirus (AgseGV) (*ix*) appear to have independently acquired their homologs from unknown ancestors. Importantly, we considered HGT of *dut* and *tmk* homologs when either widely disparate taxonomic groups or different organisms (*e.g.* alphabaculoviruses and betabaculoviruses, or group I and group II alphabaculoviruses, fugus, insects, mitochondrial genes) occur in the same clade.

The *tmk* genes are found in three different manners in the baculovirus genomes: fused to either a *polynucleotide kinase 3′-phosphatase* (*pnk*, previously annotated as a *nicotinamide riboside kinase* 1, *nrk-1*) or *dut*, or alone (Fig. 4C). In group II alphabaculoviruses, the gene is usually fused to the N-terminal portion of *pnk* (closed square/diamond, Fig. 4C). The unique exception was in ClbiNPV, where no *pnk* is found. Therefore, we concluded that some species lost the *tmk* gene during evolution (open square/diamond, Fig. 4C) and reacquired it independently from an undisclosed source (*e.g.* ClbiNPV and PeluSNPV) (Fig. 4C). On the other hand, only in PeluSNPV, ErelGV, and OpMNPV was a *tmk* gene found fused to the N-terminal region of a *dut* gene (square/circle, Fig. 4C). Finally, *tmk* was found with no fusion in most betabaculoviruses (single square, Fig. 4C).

### Two *tmk*-*dut* genes were expressed and localized distinctly in infected cells

**Figure 5:**
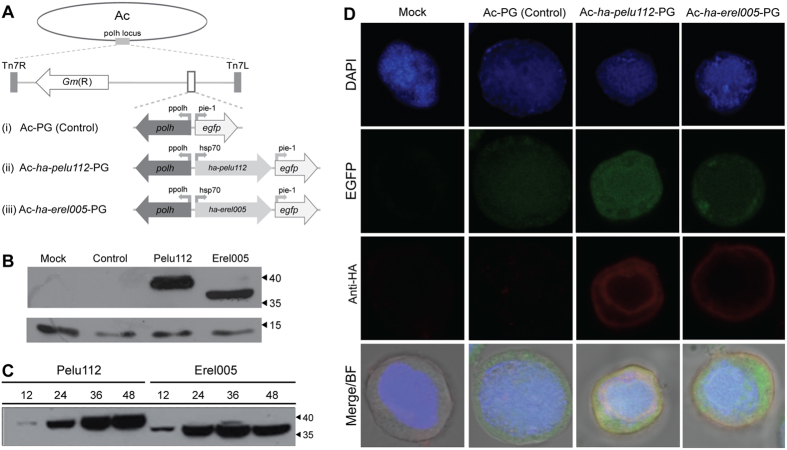
Schematic representation of engineered recombinant viruses, expression of HA-Pelu112 and HA-Erel005 proteins, and cytolocalization analyses. (**A**) The HA-tagged genes were inserted into the AcMNPV genome under the control of an insect constitutive promoter (hsp70). (**B**) Cells were mock-infected or infected with (i) Ac-PG (Control), (ii) Ac-*ha-pelu112*-PG (Pelu), or (iii) Ac-*ha-erel005*-PG (Erel) at an MOI of 0.01. Cells were harvested at 48 h p.i., and the proteins were analyzed by immunoblotting with anti-HA antibody. An over-exposure-derived unspecific reactive band is shown as a loading control. (**C**) Expression kinetics of HA-tagged proteins were assessed by immunoblotting. (**D**) Cytolocalization in virus-infected Sf9 cells. Images of virus-infected cells (MOI of 10) were photographed at 24 h p.i. using confocal laser scanning microscopy. Image panels show the red (anti-HA secondary antibody), green (GFP expressed by all recombinant viruses), and blue (DAPI) fluorescent channels. Overlays of all channels and the bright-field images are also shown (MERGE/BF).

We engineered the type baculovirus, Autographa californica multiple nucleopolyhedrovirus (AcMNPV), by inserting separately either *pelu112* or *erel005* with an N-terminal HA tag (Fig. 5A). AcMNPV naturally lacks *dut*, *tmk*, and any other nucleotide metabolism genes. The genes were inserted under the transcriptional control of a constitutive insect promoter (*Drosophila melanogaster heat shock protein 70* gene promoter)^29^. Immunoblotting analysis confirmed that both *pelu112* and *erel005* were expressed as fusions and not as cleaved proteins, based on their migration. Although both proteins have similar predicted molecular masses (37.5 kDa), *pelu112* showed a product that migrated more slowly compared to *erel005* (Fig. 5B). Time course analysis of the recombinant virus infections revealed that the proteins were first detected at 12 h p.i. and accumulated during infection progression (Fig. 5C). By confocal microscopy at 24 h p.i., Pelu112 was found close to the plasma membrane and present in the cytoplasm, and the nucleus ring-zone, while Erel005 was mostly near the plasma membrane and in the cell cytoplasm (Fig. 5D).

### *tmk-dut* expression accelerated AcMNPV progeny production.

**Figure 6:**
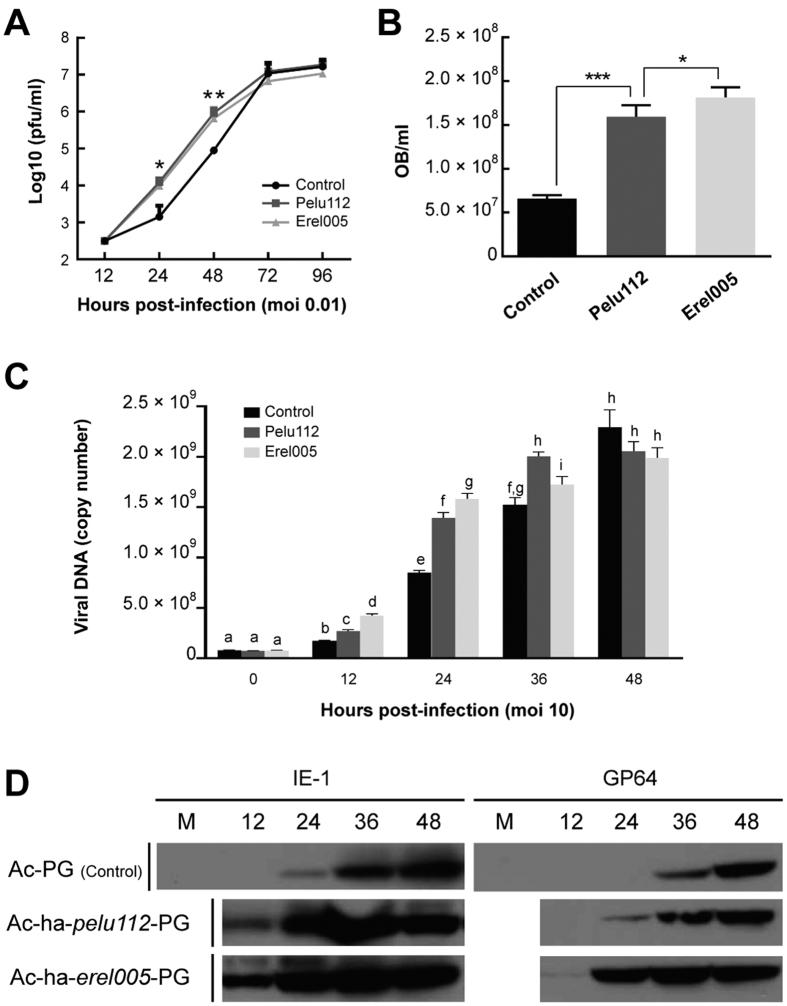
Expression of HA-Pelu112 or HA-Erel005 accelerated AcMNPV replication, viral DNA synthesis, and viral protein expression. (**A**) Analysis of BV production by endpoint dilution assays. Titers were determined from supernatants of cells infected with parental Ac-PG (Control), Ac-*ha-pelu112*-PG (Pelu), or Ac-*ha-erel005*-PG (Erel) (MOI of 0.01) at the designated time points in triplicate. Statistical differences at 24 and 48 h p.i. obtained by unpaired T-test are shown (*p* values: *p ≤ 0.01; **p ≤ 0.001). (**B**) Yields of occlusion bodies (OB) were increased 2.5-fold in the recombinant viruses. OBs were purified from Sf9 cells infected with the respective viruses (MOI of 5) at 120 h p.i. Bar heights indicate the averages of four repeats, and the error bars represent the standard deviations. Statistical differences by unpaired T-test are shown (*p* values: ***p ≤ 0.0001; *p ≤ 0.01). (**C**) Cells were infected (MOI of 10) with the indicated viruses and at 0, 12, 24, 36, and 48 h p.i. total intracellular DNA was purified and analyzed by real-time PCR in three repeats. Statistical difference by unpaired T-test are shown by letters above the bar heights. Different letters indicate that statistical difference exists. (**D**) The fused genes accelerated both IE-1 and GP64 expression during *in vitro* virus infection when compared to the control virus. Lysates obtained from the same number of cells was loaded in each lane. Cells were infected with the indicated viruses (MOI of 0.01) and at 0, 12, 24, 36, and 48 h p.i. total cellular proteins were analyzed by immunoblotting with specific anti-IE-1 or anti-GP64 antibodies.

In order to check whether expression of *pelu112* or *erel005* could influence baculovirus infection, we looked at virus progeny production *in vitro* using Sf9 cells. Interestingly, the recombinants expressing either *pelu112* or *erel005* produced higher levels of BV at 24 and 48 h p.i. than the control virus, although the final titers were similar at 72 and 96 h p.i. (Fig. 6A). For *pelu112*-expressing virus, the increase was 8.6- and 10.4-fold higher at 24 and 48 h p.i. respectively when compared to the parental virus, while for the *erel005*-expressing virus, the increase was 6.8- and 7.4-fold at the same times. Moreover, the yields of occlusion bodies (OB) were increased 2.5-fold in the *tmk*-*dut*-fused-expressing viruses compared to the control (Fig. 6B). It is important to note that in this experiment only OB production was monitored, not the ability to occlude virions.

### AcMNPV DNA replication and IE1 and GP64 expression were accelerated by expression of *tmk-dut* genes

Since homologs of *pelu112* and *erel005* are hypothetically thought to play roles in nucleotide biosynthesis pathways, we examined viral DNA replication during recombinant infection. Viral DNA replication was accelerated during recombinant infection *in vitro* and remained higher through 36 h p.i. (Fig. 6C). At 12 and 24 h p.i., the *erel005*-expressing virus produced more viral DNA than either the *pelu112*-expressing virus or the parental virus. However, at 36 h p.i. the recombinant harboring *pelu112* accumulated more DNA than the two others, while the *erel005*-expressing virus remained higher than the control. By 48 h p.i., there was no significant difference in the levels of viral DNA produced by any of the viruses. We also examined the levels of two essential virus proteins, IE-1 (the major alphabaculovirus transcription factor) and GP64 (the envelope fusion protein). Both proteins were detected earlier in cells infected with the *tmk*-*dut*-fusion-expressing viruses than with the control virus, consistent with the results observed for viral DNA replication and BV production (Fig. 6D).

### Homology modeling

**Figure 7:**
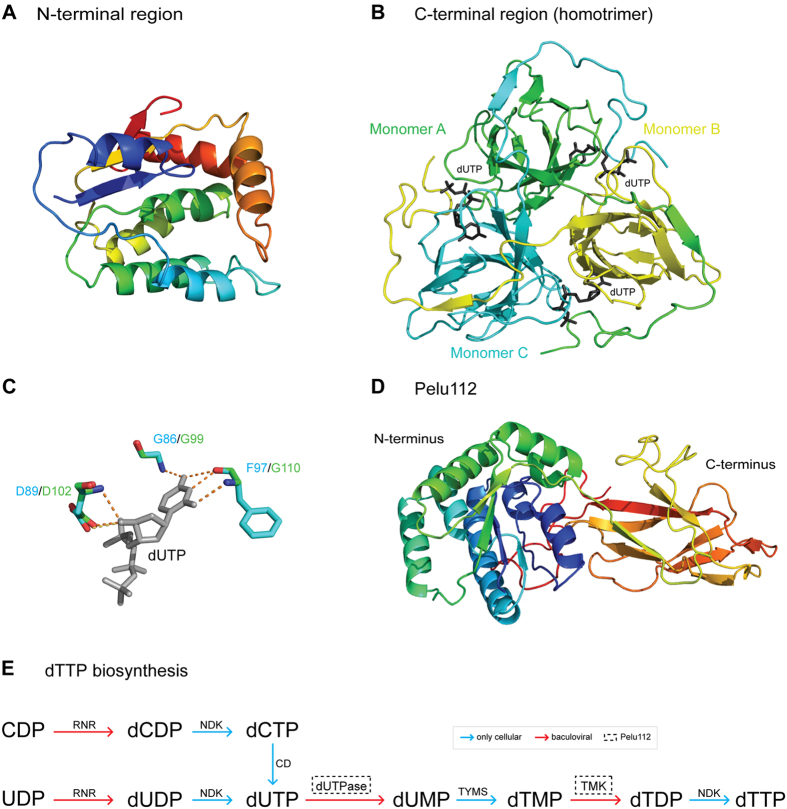
Homology modeling of Pelu112. (**A**) N-terminal region presents homology to thymidylate kinase. The model obtained presents several α-helixes as commonly is found in this enzyme. (**B**) Homotrimer model proposed for the C-terminal region of Pelu112. The three monomers interacting with their substrates (dUTP in black) are shown. (**C**) Conserved catalytic site of the modeled dUTPase interacting with dUTP (dashed lines). The template crystal used for the proposed model is shown in green overlapping the proposed model in blue. Although we identified one amino acid substitution in Pelu112 (G110 to F97), the interacting region was clearly conserved and remained stable through projecting the lateral chain to outside from the catalytic site. (**D**) Fused model of TMK-dUTPase. Both the N-terminus and C-terminus are shown. All the proposed models were constructed using previously solved protein structures available in PDB database. (**E**) The dTTP biosynthesis pathway is shown, highlighting the enzymes that are fused in Pelu112 (dashed box) and their respective actions. RNR, ribonucleotide reductase; NDK, nucleoside diphosphate kinase; CD, cytosine deaminase; TYMS, thymidylate synthase, TMK, thymidylate kinase.

In order to determine whether *pelu112* and its homologs (*op031* and *erel005*) potentially encode functional nucleotide metabolism proteins, we performed an alignment against homologs with solved crystal structures (Fig. 3). Both the viral TMK (Fig. 3A) and dUTPase (Fig. 3B) portions contained all the amino acid residues responsible for the enzymatic activity. We also built a 3D model of each domain using the predicted amino acid sequence of Pelu112. The identity between the viral sequences (N- and C terminal regions) and their homologs were 27.15% (PDB ID: 4TMK) and 28.06% (PDB ID: 3EHW), respectively. The Ramachandran plot of TMK region showed 92% residues in favored region, 5.52% in allowed region and 2.45% outliers (Fig. S2A), while the dUPTase region showed 92% residues in favored region, 6% in allowed region, and 2% outliers (Fig. S2B). The overall structure of both TMK and dUTPase homology models were similar to that of the templates. The TMK-like enzyme at the Pelu112 N-region (Fig. 7A) has an α/β fold with a three-stranded parallel β-sheet surrounded by seven α-helices, similar to other TMKs^30^. On the other hand, the Pelu112 C-terminal core, when modeled as a homotrimer, was composed of 12 β-strands (Fig. 7B and Fig. S2C). The dUTPase had homology to trimeric dUTPases and presented all five conserved motifs commonly found in trimeric dUTPases (Fig. 7B). Moreover, the N-terminal region of the monomer is projected outward leaving it free to be fused to TMK (data not shown). A fusion model is also presented in Fig. 7D and Fig. S2D. Based on this, we conclude that these motifs form a functional dUTPase active site and allow the C-terminal region of Pelu112 to form a trimeric quaternary structure with three active sites per trimer capable of interacting at the N-terminal region with other proteins (Fig. 7B). Moreover, we overlapped the catalytic site from both the template and the proposed model of the dUTPase (Fig. 7C, light green). Only one amino acid difference was observed in the catalytic site, a phenylalanine in Pelu112 rather than a glycine. Crucially, this amino acid substitution did not impact the interaction with dUTP due to the positioning of amino acid lateral chain. Therefore, it is reasonable to assume that *pelu112* encodes a *bona fide* TMK-dUTPase enzyme capable of functioning at different steps of the dTTP biosynthesis pathway (Fig. 7E).

## Discussion

The complete genome sequence of the *Perigonia lusca*-isolated group II alphabaculovirus PeluSNPV revealed that the virus is a new baculovirus species most closely related to Clanis bilineata nucleopolyhedrovirus (ClbiNPV), another sphingid-infecting virus. In the PeluSNPV genome, we found all of the 37 baculovirus core genes and many auxiliary genes including a densovirus-related non-structural homolog, *he65*-like, *chitinase*, *cathepsin*, *iap-2* and *iap-3*, and both small and large subunits of *ribonucleotide reductase*. Moreover, the genome sequence revealed a peculiar nucleotide metabolism gene acquisition (*pelu112*) which was found to be a fusion of two other genes with separate homologs in other genomes, *thymidylate kinase* or *thymidine monophosphate kinase* (*tmk*) and *dUTP diphosphatase* (*dut*), and similar fused genes seem to be acquired independently by two other distantly related baculoviruses. Reconstructing the evolutionary history of both regions of this fused gene separately, we found that this form of *tmk* seems to have been acquired several times during baculovirus evolution as a fused or non-fused protein, while the *dut* sequence has been acquired at least ten times. Furthermore, we have provided for the first time experimental evidence that expressing a fused nucleotide metabolism gene in a baculovirus that naturally lacks it resulted in accelerated *in vitro* virus progeny production, viral gene expression, and genome replication, as well as increased OB yields. Both enzymes have predicted tertiary structures, based on alignment with crystal-solved enzymes, that are similar to their cellular counterparts, which is strong, but not confirmed evidence of enzyme activity. Together, our results suggest that encoding a nucleotide metabolism gene homolog is beneficial for baculovirus replication and infection *in vitro*, and likely explains why these genes have been repeatedly acquired and retained during baculovirus evolution.

As a general rule, neither *tmk* nor *dut* are essential for baculovirus infection given that several species lack them (Fig. 4C). However, the independent and recurrent acquisition of nucleotide metabolism genes, especially *dut*, from distinct taxonomic groups by baculoviruses and other viruses strongly suggests that there is a selective advantage for viruses harboring these genes. Indeed, a gene that provides accelerated progeny production, such as that observed for the recombinant viruses produced in this study, would be probably fixed into the virus population along the course of evolution. Even though we have shown that the fused enzymes retained their individual structures and catalytic sites, our question was whether the expression of a nucleotide metabolism gene in a prototype baculovirus that naturally lacks it might change the kinetics of virus replication. Therefore, we chose to study this fused gene for two main reasons; firstly, the gene has been independently acquired three times during baculovirus evolution and secondly, the gene is a fusion of two nucleotide metabolism genes, and thus the contribution of both genes to fitness can be addressed simultaneously.

In an attempt to understand and explain our results, we found in previously published work that the expression of cellular dUTPase is regulated by the cell cycle and is at higher levels in dividing cells than in non-dividing cells^31^,^32^. In the context of virus infection, uracil incorporation controlled by the expression of cellular dUTPase and enzymes related to dTTP biosynthesis could work as a weapon against viruses^33^. An advantage for viruses to be able to replicate efficiently in a heterogeneous cell type tissue may allow them to establish infection more effectively in the host^31^,^32^. Recent work indicates that viral dUTPase activity is important in non-replicating host cells, where the dUTP/dTTP ratio is high^34^. Infection of non-replicating cells can result in progeny genomes containing dUTP, which can lead virus attenuation or direct damage to viral genomes by host repair machinery. On the other hand, viral TMK activity, by causing dUTP hydrolysis, can also reduce dUTP/dTTP ratios. Therefore, a virus that harbors dUTPase, TMK, and other enzymes related to nucleotide metabolism (*e.g.* ribonucleotide reductase) may replicate more efficiently in cells that are not in S phase by reducing dUTP concentration. In the case of several viruses including herpersviruses, asfavirus, and several lentiviruses, dUTPase activity is not necessary for replication in dividing cells, but in non-dividing cells virus replication is significantly reduced when dUTPase activity is low^35^,^36^.

It may seem unclear why expression of the viral TMK-dUTPase would improve AcMNPV replication in an actively dividing cell line like Sf9, which is likely to have a low dUTP/dTTP ratio. However, insect cells do not undergo synchronous division when cultivated *in vitro* as stable lineages^35^,^36^ and hence the dUTP/dTTP ratios likely varies between cells. Consistent with this, it has been shown that a lepidopteran cell culture with higher percentages of cells in middle and late S phase (with likely low dUTP/dTTP ratio) are more susceptible to baculovirus infection than cultures inoculated with virus in the G_2_ phase^36^. Related to this, in the case of the four known betabaculoviruses that harbor nucleotide metabolism genes (*e.g. dut*, *tmk*, or *rnr*), each species possesses a *dut* gene that appears to have been captured on four independent occasions. Both AgseGV and EpapGV are known to present polyorganotropic pathology^37^,^38^,^39^,^40^,^41^,^42^,^43^ which means that the virus can spread throughout the insect body and is not restricted to the midgut. Since cell division rates vary according to the tissue type, nucleotide metabolism genes could help viruses overcome the non-dividing cell state of some tissues. Interestingly, EpapGV codes for a novel enzyme TMK^44^ that seems to differ from the *tmk* gene in PeluSNPV. We did not find a close relationship by BLAST search between them; therefore, the *tmk* gene found at the N-terminal region of Pelu112 has no clearly defined source. TMK enzymes have been found in several viruses from different families including *Asfaviridae*, *Herpesviridae*, and *Poxviridae*. In vaccinia virus, the enzyme was not essential for virus replication but was able to complement the enzyme of a *Saccharomyces cerevisae tmk* mutant^45^.

Overall, we have shown that expression of a *tmk-dut* fusion accelerates the replication of a baculovirus. Both *tmk* and *dut* gene acquisition has happened independently several times during baculovirus evolution, which also seems to be a common feature among other viruses (*e.g.* herpesvirus, iridovirus, phycodnavirus, adenovirus, and lentivirus). Our results have presented the first clues for explaining nucleotide metabolism gene fixation in baculovirus genomes. Overall, in this context, the identification and sequencing of novel virus species or isolates, especially from countries with high diversity of flora and fauna such as Brazil, has provided a wider empirical database to help understand baculovirus evolution.

## Material and Methods

### Virus purification

Insect cadavers of *P. lusca ilus* with symptoms of baculovirus infection were collected in mate tea crops. The cadavers were kept frozen until used for further OB purification^46^. Briefly, the insect cadavers were homogenized with the same volume of ddH2O (w/v), filtered through gauze and centrifuged at 7,000 × g for 10 min. The supernatant was discarded and the pellet was resuspended in the same volume of 0.5% SDS and centrifuged at 7,000 × g for 10 min. This procedure was repeated twice. The final pellet was resuspended in 0.5 M NaCl, centrifuged once more as above, and resuspended with ddH2O. The suspended solution was loaded onto a discontinuous sucrose gradient (40-80% sucrose in Phosphate Buffered Saline [PBS], 137 mM NaCl, 2.7 mM KCl, 10.0 mM Na_2_HPO_4_, 2.0 mM KH_2_PO_4_, pH 7.4) and centrifuged at 130,000 × g for 3 h. The band containing the OBs was removed from the gradient, diluted five-fold with ddH2O, and centrifuged at 7,000 × g for 10 min.

### Scanning electron microscopy (SEM) and genomic DNA restriction analyses

One hundred μl of the OB-containing suspension (10^9^ OBs/ml of ddH_2_O) were used for SEM according to a previously published protocol^47^. For endonuclease restriction analyses, OBs were dissolved in alkaline solution and used to extract DNA^46^. Both the quantity and quality of the purified DNA were determined by electrophoresis on a 0.8% agarose gel (data not shown). The viral DNA (1–2 μg) was individually cleaved with the restriction enzymes *Apa*I, *BamH*I, *Pst*I, *Xba*I, *Xho*I, *Bgl*II, *Nsi*I, or *Cla*I (Promega) according to manufacturer’s instructions. The DNA fragments were resolved by 0.8% agarose gel electrophoresis^48^, visualized, and photographed in AlphaImager^®^ Mini (Alpha Innotech).

### Genome sequencing, assembly, and annotation

PeluSNPV genomic DNA was sequenced with a 454 Genome Sequencer (GS) FLX™ Standard (Roche) at the ‘Centro de Genômica de Alto Desempenho do Distrito Federal’ (Center of High-Performance Genomic, Brasilia, Brazil). The genome was assembled *de novo* using Geneious 7.0 (http://www.geneious.com)^49^ and the *in silico*-predicted restriction enzyme digestion profile was compared to the profiles presented in the Fig. S1B. One homologous region with low coverage was amplified (PeluOrf-7 F GGG TCA TAC ATC GTA TCA CCA AGC G and Pelu-p74 R CAT CTT ATC GGT TGG CGT ACG TGA C), cloned into pCRII (Invitrogen), and sequenced by the Sanger method (GENEWIZ^®^, Inc., USA). The open reading frames (ORFs) that started with a methionine codon (ATG) and encoded polypeptides of at least 50 amino acids were identified with Genious 7.0^49^ and annotated using BLASTP^50^. We considered an acceptable overlap of less than 50% of the ORF residing within the neighbor ORFs. Both Tandem Repeats Finder (http://tandem.bu.edu/trf/trf.html)^51^ and Geneious 7.0^49^ programs were used to locate homologous regions (*hrs*) and direct repeats (*drs*).

### Phylogenetic analyses

For *Baculoviridae* phylogenetic analysis, a MAFFT alignment^52^ was carried out with concatenated amino acid sequences of 37 baculoviral core genes from 73 baculovirus genomes publicly available (Table S2). A maximum likelihood tree was inferred using a MAFFT alignment, the Fast-tree method^53^ and a Shimodaira-Hasegawa-like test^54^. Horizontal gene transfer (HGT) events were investigated using the same method described above. MAFFT alignments (available upon request) of 36 sequences (for the *cp016*-like genes) and 88 sequences (for *dut* genes) of homologs were used with the multiple sequence alignment package T-Coffee^55^. Both the tree for *cp016*-like and *dut* gene were transformed to a cladogram using FigTree v1.4.0. All the alignments are available upon request.

### Viruses and insect cell line

*Spodoptera frugiperda* (fall armyworm) (Sf9) cells^56^ were maintained at 27 °C in TC-100 medium (Invitrogen), supplemented with 10% fetal bovine serum (FBS, Invitrogen), penicillin G (60 μg/ml), streptomycin sulfate (200 μg/ml), and amphotericin B (0.5 μg/ml). Recombinant AcMNPV-C6 were propagated in insect cell cultures and their titers determined by end-point dilution^46^.

### Gene amplification, shuttle vectors, and recombinant AcMNPV virus construction

Gene from PeluSNPV (*pelu112*) and ErelGV (*erel005*) were separately amplified using two set of primers (Pelu F - ACA ACAGAG CTC ATG AAG ACC TAC ATT TGT GGT AC and Pelu R - AAT AGC GGC CGC TTA AAA AGT AGA TCC GAA TC, Erel F - ACA ACAGAG CTC ATG AAG ACC TAC ATT TGC GGT ACG and Erel R - AAA CGC GG CCG CTT AAG AAG TAG ACC CGA ACC) in two reactions which contained 100 ng of the DNA-template (PeluSNPV or ErelGV genomes), 300 μM of dNTP mix (Fermentas, Pittsburgh, PA, USA), 0.4 μM of each set of primer pairs, 1 U of VENT Polymerase (New England Biolabs, Ipswich, MA, USA), and 1x of the supplied reaction buffer. The reactions were subjected to the following program: 95 °C/2 min, 35 cycles of 95 °C/ 30 s, 55 °C/30 s and 68 °C/1 min with a final extension of 5 min at 68 °C. The amplified fragments were digested with *Sac*I/*Not*I (New England Biolabs, Ipswich, MA, USA) and cloned into pFB-PG-H-ha-pA shuttle plasmid (a modified pFB-PG containing a SV40-polyA signal and the *Drosophila melanogaster* hsp70 promoter to drive the heterolog gene expression with a for-fusion-ha-tag before the restriction sites)^29^ and confirmed by restriction digestion and sequencing (GENEWIZ^®^, Inc., USA). The modified plasmids containing the heterologous genes were transformed into DH10-Bac cells (Invitrogen, Carlsbad, CA, USA) by heat shock^48^. Recombinant bacmids were selected and confirmed by PCR following the manufacturer’s instructions (Bac-to-Bac^®^, Baculovirus expression systems, Invitrogen, Carlsbad, CA, USA). One μg of each recombinant bacmid was transfected into Sf9 cells (10^6^) using Lipofectin^57^. The supernatant of seven day post-transfection cells containing the recombinant viruses were collected, amplified in Sf9 cells, and titered as previously described^46^.

### Immunofluorescence

Sf9 cells (1 × 10^6^) were seeded on coverslips in 35-mm-diameter culture dishes and infected at MOI of 10 with recombinant viruses. At 24 h p.i., the supernatant was removed and the cells were washed twice with PBS, pH 6.2, and fixed in 2.5% formaldehyde in PBS for 10 min at room temperature (RT). The fixed cells were washed three times in PBS for 5 min, followed by permeabilization in 0.1% NP-40 (Sigma) in PBS for 10 min at RT. Cells were washed three times in PBS for 5 min per wash before incubation with blocking solution (5% BSA, 0.3% Triton-100 in PBS) for 1 h at RT, followed by incubation with anti-HA (1:500) in PBS with 1% BSA, 0.3% Triton X-100 overnight at 4 C in a humid chamber. Cells were washed three times in blocking solution for 5 min each, followed by 1 h incubation with Alexa Fluor 594-conjugated goat anti-rabbit antibody (1:1,000) in the dark at RT. Cells were washed three times for 5 min each in PBS, followed by incubation with DAPI (Invitrogen) solution according to the manufacture instructions in PBS for 15 min at RT. The cells were subsequently washed three times for 10 min each in PBS. Coverslips were mounted on a glass slide with Fluoromount-G (Southern Biotech) and stored at 4 °C in the dark until examined with a Carl Zeiss LSM 5 Pascal Laser Scanning Confocal Microscope.

### Virus growth curves and polyhedra production

For viral growth curve analyses, three independent Sf9 cell dishes (0.5 × 10^6^ cells/35-mm-diameter dish) were infected (MOI of 0.01 TCID_50_/cell) for 1 h and then washed twice with TC-100 medium and replenished with 2 ml of fresh TC-100 medium supplemented with 10% FBS. The supernatants of the infected cells were collected at various time points to determine titers by 50% tissue culture infective dose (TCID50) endpoint dilution assays ^46^on Sf9 cells. For polyhedra production, three independent infections were separately performed in Sf9 cells at 80% confluency in cell culture flasks (75 cm^2^) at MOI of 5 TCID_50_/cell. Cell monolayers were incubated for 1 h with the virus inocula, washed twice with TC-100 medium, and replenished with 12 ml fresh TC-100 medium supplemented with 10% FBS. The cells and polyhedra released were collected at 120 h p.i. and purified according to previously published protocol^46^. The purified OBs were diluted in the same volume, homogenized by vortexing overnight at 200 rpm, and counted using a hemocytometer.

### Immunoblotting

Protein samples were mixed with equal volumes of 2x protein loading buffer (0.25 M Tris-Cl, pH 6.8, 4% SDS, 20% glycerol, 10% 2-mercaptoethanol, and 0.02% bromophenol blue) and incubated at 100 °C for 5 min. Samples were resolved by 15 or 12% SDS-PAGE, transferred onto polyvinylidenefluoride (PVDF) membrane (Millipore), and probed with (i) mouse monoclonal anti-hemagglutinin (anti-HA) antibody (Covance), (ii) mouse monoclonal anti-GP64 antibody (eBioscience), or (iii) mouse polyclonal anti-IE-1 antibody; this probing was followed by incubation with horseradish peroxidase-conjugated secondary antibodies (Sigma). Blots were developed using the Super Signal West Pico chemiluminescent substrate (Pierce) and exposed to X-ray films.

### Quantitative real-time PCR (Q-PCR)

To detect viral DNA replication in virus-infected cells, Q-PCR was performed as previously described. Sf9 cells (1.0 × 10^6^ cells/35-mm-diameter dish) were infected in triplicate at MOI of 5 TCID_50_/cell, and cells were collected at different time points. Total DNA was prepared with the Wizard genomic DNA purification kit (Promega) according to the protocol of the manufacturer. Purified DNA was quantified by optical density measurement. Q-PCR was performed with 10 ng DNA and Absolute Q-PCR SYBR green fluorescein mix (Thermo Scientific) according to the protocol of the manufacturer by using the same primers to amplify a 100-bp region of the AcMNPV *gp41* gene as described previously^58^. Standard DNA samples were used from purified AcMNPV BV DNA and serially diluted to 100, 10, 1, 0.1, 0.01, and 0.001 ng. Genomic equivalents of DNA samples were determined by extrapolation from standard curves. A melting-curve analysis of each amplified sample was carried out to check the specificity of each reaction. The results were analyzed using GraphPad Prism version 5.01 (GraphPad Software, Inc.).

### Homology modeling

The templates for three dimensional (3D) structure prediction of Pelu112 protein were searched in expasy SWISS-MODEL server^59^ using the amino acid (aa) sequence as the reference. Both models, 4TMK and 3EHW, were obtained from crystallized functional enzymes. 4TMK is a complex of *E. coli* thymidylate kinase with the bisubstrate inhibitor TP5A and 3EHW is the Human trimeric dUTPase in complex with alpha, beta-imido-dUTP and Mg^2+^. The Suitable templates were aligned with Pelu112 protein using T-Coffee server^55^ and the resulting alignments were manually improved using BioEdit^60^. Aligned sequences were used with MODELLER v. 9.10^61^ to develop high quality 3D models using the default parameters. The highest quality models were selected and the accuracy of these predicted models was further analyzed through MolProbity^62^. The validation of all these models was done by checking the psi/phi ratio of Ramachandran plot obtained from MolProbity analysis. Yasara^63^ was also applied for final models to check for energy minimization criteria. Ramachandran outlier residues were fixed with COOT^64^ and energy minimization. The models were visualized using The PyMOL molecular graphics system v. 1.0 (DeLano Scientific, San Carlos, CA).

## Additional Information

**How to cite this article**: Ardisson-Araújo, D. M. P. *et al.* Genome sequence of Perigonia lusca single nucleopolyhedrovirus: insights into the evolution of a nucleotide metabolism enzyme in the family *Baculoviridae.* *Sci. Rep.* **6**, 24612; doi: 10.1038/srep24612 (2016).

## Supplementary Material

Supplementary Information
