# Supplementary material for: Genome sequence of Perigonia lusca single nucleopolyhedrovirus: insights into the evolution of a nucleotide metabolism enzyme in the family Baculoviridae
Source: Sci Rep. 2016 Jun 7;6:24612. doi: 10.1038/srep24612 (PMC4895240; doi:10.1038/srep24612)
Supplement: Supplementary Information [file srep24612-s1.pdf]

# Genome sequence of Perigonia lusca single nucleopolyhedrovirus: insights into the evolution of a nucleotide metabolism enzyme in the family *Baculoviridae*

Daniel M. P. Ardisson-Araújo[1,2], Rayane Nunes Lima[1], Fernando L. Melo[1], Rollie J. Clem[2], Ning Huang[2], Sônia Nair Báo[1], Daniel R. Sosa-Gómez[3], Bergmann M. Ribeiro[1*]

[1]Laboratory of Baculovirus, Cell Biology Department, University of Brasília, Brasília, DF, Brazil; [2]Division of Biology, Kansas State University, Manhattan, KS, USA; [3]Embrapa Soja, Londrina, PR, Brazil.

*Corresponding author:
Dr. Bergmann M. Ribeiro
Laboratory of Baculovirus
Cell Biology Department
University of Brasilia
Brasilia, DF, 70910-900
tel: 55-61-31070478
email: bergmann@unb.br

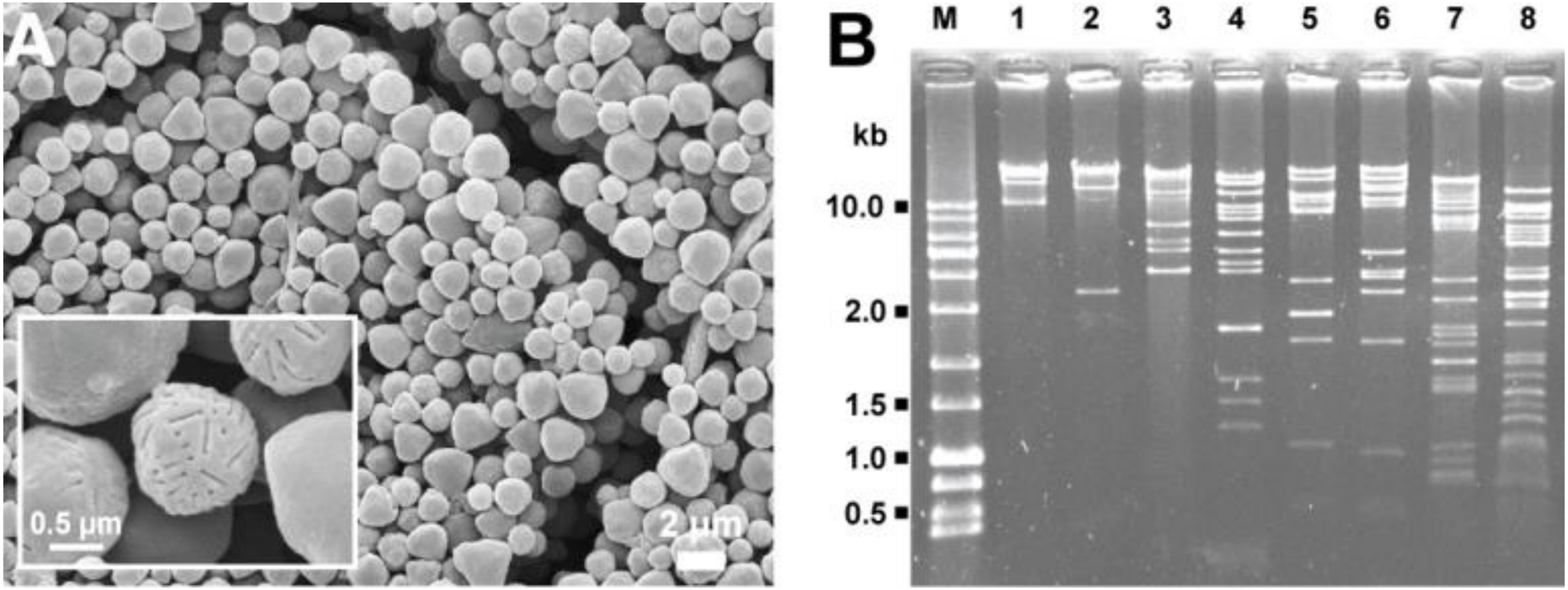


Figure S1.Structural analyses of PeluSNPV. (A) Scanning electron microscopy of purified polyhedral occlusion bodies (OBs) of irregular shape and size. Immature OBs are inset. Moreover, indentation that presumably contained singly embedded rod-shaped nucleocapsids are visible lost during sample preparation. (B) Agarose gel electrophoresis-resolved DNA fragments digested with each *Apa*I (lane 1), *Bam*HI (lane 2), *Pst*I (lane 3), *Xba*I (lane 4), *Xho*I (lane 5), *Bgl*II (lane 6), *Nsi*I (lane 7), or *Cla*I (lane 8). Molecular mass marker (lane M).

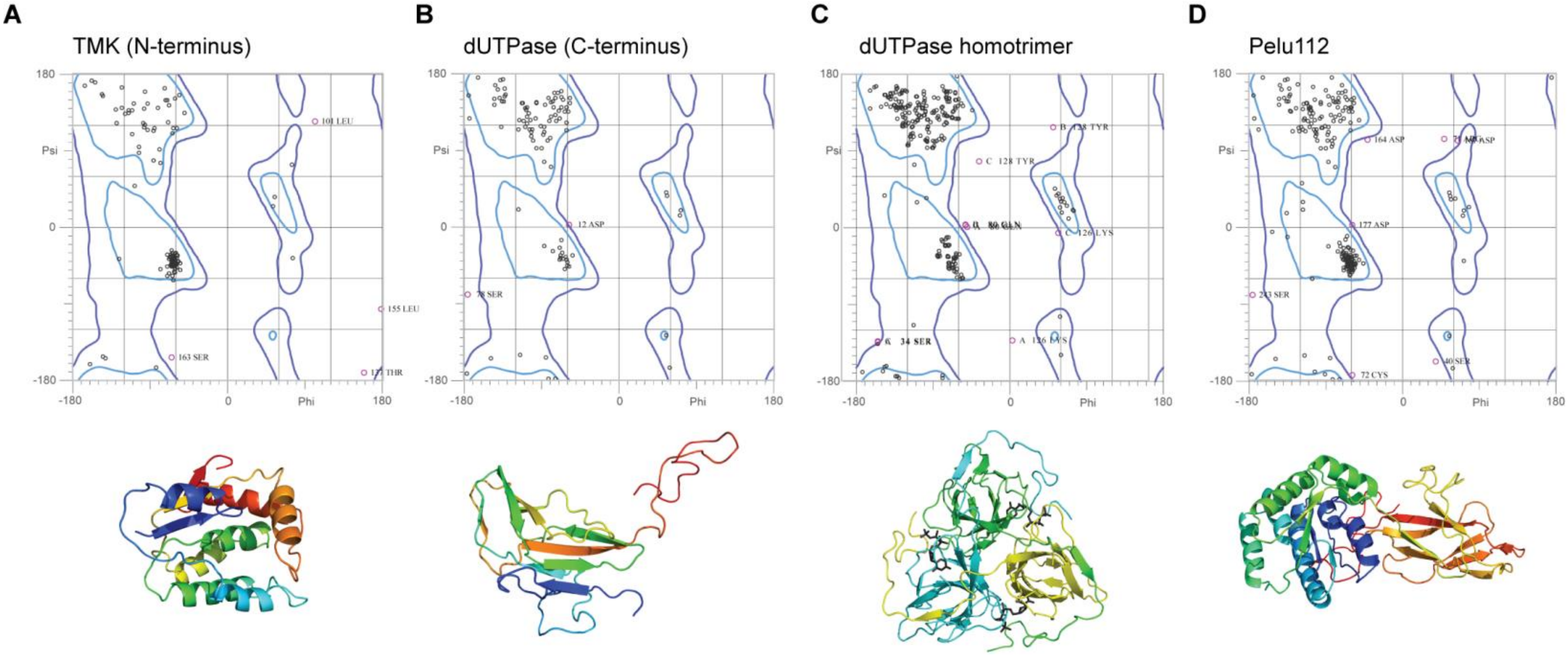


Figure S2. Ramachandran plot for each proposed protein model. (A) N-terminal region of Pelu112, the TMK-like enzyme. (B) C-terminal region of Pelu112, the dUTPase-like enzyme. (C) dUTPase homotrimer. (C) Fused model. The individual, assembled, or fused structures are shown below each respective plot.

**Table S1**. Characteristics of the Perigonia lusca single nucleopolyhedrovirus (PeluSNPV) genome: analysis and homology search. Predicted ORFs are compared with homolog genes in two related genomes.

| Orf | Name | Position | Size (nt) | Size (aa) | ClbiNPV | | AcMNPV | | Best hit |
|---|---|---|---|---|---|---|---|---|---|
| | | | | | Orf | Max Id (%)[+] | Orf | Max Id (%)[+] | |
| 1 | *polh* | 1 > 741 | 741 | 246 | 1 | 91 | 8 | 89 | OrleNPV |
| 2 | *orf1629* | 902 < 2,566 | 1,665 | 554 | 2 | 27 | 9 | 29 | ClbiNPV |
| 3 | *pk-1* | 2,559 > 3,356 | 798 | 265 | 3 | 55 | 10 | 44 | AgseNPV-B |
| 4[a] | | 3,629 < 4,042 | 414 | 137 | - | - | - | - | *Ceriporiopsis subvermispora* |
| 5 | *hoar* | 4,128 < 6,239 | 2,112 | 703 | 4 | 30 | | | EcobNPV |
| 6[a] | | 6,469 > 6,657 | 189 | 62 | - | - | - | - | *Daphnia pulex* |
| 7[a] | | 6,804 > 8,276 | 1,473 | 490 | - | - | - | - | *Megasphaera sp.* |
| | *direct repeat* | 8,535 - 9,015 | 480 | - | - | - | - | - | - |
| 8 | *p74* | 9,382 < 11,358 | 1,977 | 658 | 14 | 62 | 138 | 59 | OrleNPV |
| 9 | *me53* | 11,452 < 12,522 | 1,071 | 356 | 12 | 49 | 139 | 23 | ClbiNPV |
| 10[a] | | 12,561 > 12,713 | 153 | 50 | - | - | - | - | *Beta vulgaris* |
| 11 | *ie-0* | 12,860 > 13,693 | 834 | 277 | 11 | 40 | 141 | 28 | ChchNPV |
| 12 | *p49* | 13,742 > 15,265 | 1,524 | 507 | 10 | 71 | 142 | 51 | ClbiNPV |
| 13 | *odv-e18* | 15,210 > 15,470 | 261 | 86 | 10b* | 71[§] | 143 | 83 | LyxyMNPV |
| 14 | *odv-e27* | 15,508 > 16,371 | 864 | 287 | 9 | 67 | 144 | 49 | OrleNPV |

| | | | | | | | | | | | |
|---|---|---|---|---|---|---|---|---|---|---|---|
| 15 | *chtb-1* | 16,388 | > | 16,669 | 282 | 93 | 9b* | 69[§] | 145 | 49 | AdhoNPV |
| 16 | *ep23* | 16,680 | < | 17,300 | 621 | 206 | 8 | 34 | 146 | 33 | ApciNPV |
| 17 [a] | | 17,380 | > | 17,838 | 459 | 152 | - | - | - | - | *no hit* |
| 18 [a] | | 17,901 | > | 18,380 | 480 | 159 | - | - | - | - | no hit |
| 19 | *ie-1* | 18,245 | > | 19,615 | 1,371 | 456 | 7 | 41 | 147 | 31 | EcobNPV |
| 20 | *odv-e56 (pif-5)* | 19,769 | < | 20,836 | 1,068 | 355 | 6 | 61 | 148 | 54 | OrleNPV |
| | *hr-1* | 21,131 | - | 21,897 | 766 | - | - | - | - | - | - |
| 21 | *p47* | 22,004 | > | 23,218 | 1,215 | 404 | 36 | 65 | 40 | 54 | HespNPV |
| 22 | *dbp-1* | 23,378 | < | 24,301 | 924 | 307 | 27 | 46 | 25 | 29 | ClbiNPV |
| 23 | *nudix; bv-e31* | 24,479 | > | 25,354 | 876 | 291 | 25 | 57 | 38 | 52 | AgipMNPV |
| 24 | *lef-11* | 25,198 | > | 25,800 | 603 | 200 | - | - | 37 | 34 | AgseNPV-B |
| 25 | *39k* | 25,736 | > | 26,689 | 954 | 317 | 24 | 43 | 36 | 37 | ClbiNPV |
| 26 [a] | | 26,833 | < | 27,012 | 180 | 59 | - | - | - | - | no hit |
| 27 | *v-ubq* | 26,969 | < | 27,247 | 279 | 92 | 22 | 80 | 35 | 76 | HespNPV |
| 28 | *lef-7* | 27,414 | > | 28,388 | 975 | 324 | 37 | 39 | 125 | 31 | MaviNPV |
| 29 | | 28,404 | > | 29,021 | 618 | 205 | 21 | 54 | 34 | 33 | HespNPV |
| 30 | *p10* | 29,152 | < | 29,415 | 264 | 87 | 20 | 63 | 137 | 29 | ChchNPV |
| 31 | *p26-1* | 29,517 | < | 30,377 | 861 | 286 | 19 | 42 | 136 | 32 | ApciNPV |
| 32 | | 30,583 | > | 30,834 | 252 | 83 | 18 | 48 | 29 | 29 | MacoNPV-A |
| 33 | *lef-6* | 30,963 | < | 31,733 | 771 | 256 | 17 | 55 | 28 | 47 | AgseNPV |

| | | | | | | | | | | | |
|---|---|---|---|---|---|---|---|---|---|---|---|
| 34 | *dbp-2* | 31,781 | < | 32,560 | 780 | 259 | 16 | 36 | 25 | 30 | AgseNPV |
| 35 [a] | | 32,780 | > | 32,938 | 159 | 52 | - | - | - | - | *Saccharomonospora viridis* |
| 36 | *lef-12* | 33,316 | > | 34,035 | 720 | 239 | 34 | 28 | 41 | 37 | AdorNPV |
| 37 | | 34,025 | > | 34,276 | 252 | 83 | 33 | 38 | 43 | 31 | BomaNPV |
| 38 | | 34,295 | < | 34,843 | 549 | 182 | - | - | - | - | MacoNPV-A |
| 39 | *ctl-1* | 34,946 | > | 35,131 | 186 | 61 | 53 | 60 | 3 | 40 | ChmuNPV |
| 40 | *lef-9* | 35,207 | < | 36,703 | 1,497 | 498 | 47 | 76 | 62 | 68 | ChchNPV |
| 41 | *fp-25k* | 36,892 | > | 37,536 | 645 | 214 | 46 | 67 | 61 | 60 | OrleNPV |
| 42 | *bro-a* | 37,725 | > | 38,108 | 384 | 127 | 105 | 52 | - | - | HespNPV |
| 43 | *chab-a* | 38,202 | > | 38,516 | 315 | 104 | 45 | 56 | 60 | 56 | LdMNPV |
| 44 | *chab-b* | 38,568 | > | 39,068 | 501 | 166 | 44 | 72 | 58/59 | 47 | MacoNPV-A |
| 45 | | 39,121 | < | 39,693 | 573 | 190 | 43 | 42 | 57 | 43 | BusuNPV |
| 46 | | 40,084 | < | 40,338 | 255 | 84 | - | - | - | - | AdorNPV |
| 47 | | 40,277 | < | 40,564 | 288 | 95 | 41 | 59 | 55 | 40 | SujuNPV |
| 48 [a] | | 40,545 | > | 40,751 | 207 | 68 | - | - | - | - | *no hit* |
| 49 | *vp1054* | 40,685 | < | 41,749 | 1,065 | 354 | 39 | 48 | 54 | 39 | AgipMNPV |
| 50 | *lef-10* | 41,604 | < | 41,837 | 234 | 77 | - | - | 53a | 33 | TnSNPV |
| 51 | | 41,800 | > | 42,030 | 231 | 76 | - | - | - | - | TnSNPV |
| 52 | | 42,047 | > | 43,141 | 1,095 | 364 | 38 | 31 | - | - | HespNPV |
| 53 | | 43,130 | < | 43,555 | 426 | 141 | 28 | 63 | 53 | 46 | OrleNPV |

| | | | | | | | | | | | |
|---|---|---|---|---|---|---|---|---|---|---|---|
| 54 [a] | | 43,613 | > | 43,858 | 246 | 81 | - | - | - | - | *no hit* |
| 55 [a] | | 43,708 | < | 43,965 | 258 | 85 | - | - | - | - | *Flavobacterium soli* |
| 56 | *dnaj* | 43,990 | < | 44,886 | 897 | 298 | 31 | 34 | - | - | ClbiNPV |
| 57 | *lef-8* | 44,907 | > | 47,582 | 2,676 | 891 | 32 | 69 | 50 | 62 | ApciNPV |
| 58 | *gp37* | 47,791 | < | 48,741 | 951 | 316 | 56 | 57 | 64 | 47 | ClbiNPV |
| 59 | | 48,918 | < | 49,124 | 207 | 68 | 58 | 46 | 111 | 52 | BmNPV |
| 60 | *chitinase* | 49,277 | < | 50,989 | 1,713 | 570 | 59 | 72 | 126 | 71 | ClbiNPV |
| 61 | *v-cath* | 51,109 | > | 52,122 | 1,014 | 337 | 60 | 69 | 127 | 69 | SujuNPV |
| 62 | *p26-2* | 52,171 | < | 52,899 | 729 | 242 | 61 | 44 | 136 | 28 | ClbiNPV |
| 63 | *chtB-2* | 52,992 | < | 53,330 | 339 | 112 | 62 | 45 | 150 | 31 | HaNPV |
| 64 | *iap-2* | 53,334 | < | 54,077 | 744 | 247 | 63 | 34 | 71 | 31 | AgseNPV |
| 65 | *mtase-1* | 54,074 | < | 54,886 | 813 | 270 | 64 | 52 | 69 | 49 | SpliNPV-II |
| 66 | | 54,858 | < | 55,232 | 375 | 124 | - | - | 68 | 41 | AgseNPV |
| 67 | *lef-3* | 55,394 | > | 56,470 | 1,077 | 358 | 65 | 43 | 67 | 28 | ClbiNPV |
| 68 | *desmoplakin* | 56,641 | < | 58,983 | 2,343 | 780 | 66 | 32 | 66 | 31 | TnSNPV |
| 69 | *dna-pol* | 59,021 | > | 62,206 | 3,186 | 1061 | 67 | 65 | 65 | 48 | ClbiNPV |
| 70 | | 62,300 | < | 62,689 | 390 | 129 | 68 | 60 | 75 | 31 | ClbiNPV |
| 71 | | 62,697 | < | 62,954 | 258 | 85 | 69 | 72 | 76 | 59 | OrleNPV |
| 72 | *vlf-1* | 63,029 | < | 64,207 | 1,179 | 392 | 71 | 81 | 77 | 73 | ClbiNPV |
| 73 | | 64,219 | < | 64,569 | 351 | 116 | 72 | 72 | 78 | 59 | BusuNPV |

| | | | | | | | | | | | |
|---|---|---|---|---|---|---|---|---|---|---|---|
| 74 | *gp41* | 64,640 | < | 65,602 | 963 | 320 | 73 | 79 | 80 | 60 | ClbiNPV |
| 75 | | 65,729 | < | 66,247 | 519 | 172 | 74 | 57 | 81 | 60 | TnSNPV |
| 76 | *tlp20* | 66,177 | < | 66,938 | 762 | 253 | 75 | 48 | 82 | 35 | EupsNPV |
| 77 | *p95 (vp91)* | 66,808 | > | 69,255 | 2,448 | 815 | 76 | 39 | 83 | 35 | ApciNPV |
| 78 | *cg30* | 69,491 | < | 70,228 | 738 | 245 | 77 | 32 | 88 | 32 | OrleNPV |
| 79 | *vp39* | 70,330 | < | 71,337 | 1,008 | 335 | 78 | 58 | 89 | 40 | ClbiNPV |
| 80 | *lef-4* | 71,336 | > | 72,886 | 1,551 | 516 | 79 | 53 | 90 | 45 | HespNPV |
| 81 | *p33 (sox)* | 72,916 | < | 73,617 | 702 | 233 | 80 | 65 | 92 | 47 | ClbiNPV |
| 82 | *p18* | 73,696 | > | 74,196 | 501 | 166 | 81 | 66 | 93 | 48 | PespNPV |
| 83 | *odv-e25* | 74,193 | > | 74,888 | 696 | 231 | 82 | 72 | 94 | 42 | ClbiNPV |
| 84 | *dna-helicase* | 75,018 | < | 78,680 | 3,663 | 1220 | 83 | 58 | 95 | 42 | OrleNPV |
| 85 | *odv-e28 (pif-4)* | 78,649 | > | 79,173 | 525 | 174 | 84 | 61 | 96 | 50 | OrleNPV |
| 86 | *38k* | 79,214 | < | 80,254 | 1,041 | 346 | 85 | 59 | 98 | 49 | ClbiNPV |
| 87 | *lef-5* | 80,150 | > | 81,037 | 888 | 295 | 86 | 61 | 99 | 48 | OrleNPV |
| 88 | *p6.9* | 81,055 | < | 81,285 | 231 | 76 | 86b* | 44[§] | 100 | 42[§] | *no hit* |
| 89[a] | | 81,240 | > | 81,410 | 171 | 56 | - | - | - | - | *no hit* |
| 90 | *p40* | 81,347 | < | 82,522 | 1,176 | 391 | 87 | 56 | 101 | 39 | ClbiNPV |
| 91 | *p12* | 82,541 | < | 82,912 | 372 | 123 | 88 | 59 | 102 | 36 | ClbiNPV |
| 92 | *p48/p45* | 82,905 | < | 84,092 | 1,188 | 395 | 89 | 69 | 103 | 39 | ClbiNPV |
| 93 | *vp80* | 84,121 | > | 86,733 | 2,613 | 870 | 90 | 28 | 104 | 24 | ClbiNPV |

| | | | | | | | | | | | |
|---|---|---|---|---|---|---|---|---|---|---|---|
| 94 | | 86,755 | > | 86,922 | 168 | 55 | 91 | 59 | 110 | 35 | EcobNPV |
| 95 | *odv-ec43* | 86,929 | > | 88,008 | 1,080 | 359 | 92 | 72 | 109 | 43 | ClbiNPV |
| 96 | | 88,077 | > | 88,367 | 291 | 96 | - | - | - | - | SfMNPV |
| 97 | *p13* | 88,397 | < | 89,218 | 822 | 273 | 94 | 62 | - | - | SpliNPV-II |
| 98 | | 89,273 | > | 90,373 | 1,101 | 366 | 95 | 31 | 112/113 | 36 | LyxyMNPV |
| 99 [a] | | 90,556 | > | 90,906 | 351 | 116 | - | - | - | - | *Arabidopsis thaliana* |
| 100 [a] | | 90,810 | < | 91,187 | 378 | 125 | - | - | - | - | no hit |
| 101 [a] | | 91,056 | > | 91,829 | 774 | 257 | - | - | - | - | *Halomonas sp.* |
| 102 | *iap-3* | 91,830 | > | 92,432 | 603 | 200 | - | - | - | - | LdMNPV |
| 103 | | 92,443 | < | 93,135 | 693 | 230 | 97 | 92 | 106 | 64 | ClbiNPV |
| 104 | | 93,294 | > | 94,025 | 732 | 243 | - | - | - | - | ErelGV |
| 105 | *pagr* | 94,075 | < | 95,580 | 1,506 | 501 | 98 | 21 | - | - | SujuNPV |
| 106 | | 95,671 | < | 96,060 | 390 | 129 | 99 | 39 | - | - | ApciNPV |
| 107 | *pif-3* | 96,071 | < | 96,697 | 627 | 208 | 100 | 44 | 115 | 44 | SpliNPV-II |
| 108 | *sod* | 96,777 | > | 97,268 | 492 | 163 | 102 | 76 | 31 | 73 | ClbiNPV |
| 109 | | 97,317 | < | 98,336 | 1,020 | 339 | - | - | 11 | 47 | AgMNPV |
| 110 | | 98,277 | > | 98,459 | 183 | 60 | - | - | - | - | ChroNPV |
| 111 | *ctl-2* | 98,483 | > | 98,644 | 162 | 53 | - | - | 3 | 74 | AcMNPV |
| 112 | *dut-fused* | 98,827 | > | 99,780 | 954 | 317 | - | - | - | - | ErelGV |
| 113 | | 99,938 | > | 100,315 | 378 | 125 | 103 | 33 | - | - | AgseNPV |

| | | | | | | | | | | | |
|---|---|---|---|---|---|---|---|---|---|---|---|
| 114 | | 100,312 | > | 100,590 | 279 | 92 | 104 | 40 | 117 | 40 | ClbiNPV |
| 115 | *pif-2* | 100,652 | < | 101,794 | 1,143 | 380 | 107 | 72 | 22 | 66 | BusuNPV |
| 116 | *pkip* | 101,837 | < | 102,415 | 579 | 192 | 108 | 33 | - | - | HzSNPV |
| 117 | *lef-2* | 102,469 | < | 103,098 | 630 | 209 | 109 | 57 | 6 | 42 | ClbiNPV |
| 118 | | 103,070 | < | 103,438 | 369 | 122 | 110 | 43 | - | - | AdorNPV |
| 119 [a] | | 103,643 | < | 104,098 | 456 | 151 | - | - | - | - | *Sulfolobus islandicus* |
| | *hr-2* | 103,658 | - | 104,027 | 740 | | | | | | |
| 120 [a] | | 104,260 | < | 104,409 | 150 | 49 | - | - | - | - | no hit |
| 121 | *p24* | 104,462 | > | 105,241 | 780 | 259 | 111 | 54 | 129 | 40 | HespNPV |
| 122 | | 105,242 | < | 105,712 | 471 | 156 | 112 | 30 | - | - | HespNPV |
| 123 | *gp16* | 105,810 | > | 106,106 | 297 | 98 | 113 | 49 | 130 | 37 | TnSNPV |
| 124 | *he65* | 106,230 | > | 107,057 | 828 | 275 | - | - | 105 | 35 | AgseGV |
| 125 | *pep; pp34* | 107,208 | > | 108,128 | 921 | 306 | 114 | 55 | 131 | 28 | OrleNPV |
| 126 | *rr2a* | 108,210 | < | 109,262 | 1,053 | 350 | - | - | - | - | HespNPV |
| 127 | | 109,345 | < | 109,758 | 414 | 137 | 115 | 44 | 19 | 36 | OrleNPV |
| 128 | | 109,769 | > | 110,989 | 1,221 | 406 | 116 | 32 | 18 | 27 | AgseNPV |
| 129 | *alk-exo* | 111,007 | > | 112,266 | 1,260 | 419 | 117 | 48 | 133 | 39 | ApciNPV |
| 130 | | 112,341 | < | 113,060 | 720 | 239 | - | - | - | - | AgseNPV-B |
| 131 | *fgf* | 113,223 | > | 114,365 | 1,143 | 380 | 118 | 33 | 32 | 21 | SujuNPV |
| 132 | | 114,379 | < | 114,615 | 237 | 78 | - | - | - | - | AgseNPV-B |

| | | | | | | | | | | | |
|---|---|---|---|---|---|---|---|---|---|---|---|
| 133 | *pif-1* | 114,618 | < | 116,240 | 1,623 | 540 | 120 | 48 | 119 | 51 | ApciNPV |
| 134 | *odv-e66* | 116,280 | < | 118,259 | 1,980 | 659 | - | - | 46 | 45 | OrleNPV |
| 135 | *f protein* | 118,381 | < | 120,507 | 2,127 | 708 | 129 | 67 | 23 | 23 | ClbiNPV |
| 136 | | 120,663 | > | 123,596 | 2,934 | 977 | 128 | 42 | - | - | ClbiNPV |
| 137 | | 123,633 | < | 124,490 | 858 | 285 | 127 | 33 | 17 | 36 | HespNPV |
| 138 | | 124,598 | < | 125,272 | 675 | 224 | 126 | 48 | - | - | ClbiNPV |
| 139 | *egt* | 125,504 | < | 127,117 | 1,614 | 537 | 125 | 47 | 15 | 52 | AcMNPV |
| 140 [a] | | 127,192 | < | 127,401 | 210 | 69 | - | - | - | - | no hit |
| 141 | | 127,362 | < | 127,700 | 339 | 112 | 124 | 56 | - | - | OrleNPV |
| 142 | *lef-1* | 127,719 | > | 128,411 | 693 | 230 | 123 | 48 | 14 | 42 | ClbiNPV |
| 143 | *38.7k* | 128,429 | > | 129,580 | 1,152 | 383 | 122 | 39 | 13 | 41 | ClbiNPV |
| 144 [a] | | 129,678 | < | 130,292 | 615 | 204 | - | - | - | - | *Plasmodium vinckei petteri* |
| 145 | *rr1* | 130,348 | < | 132,645 | 2,298 | 765 | - | - | - | - | EupsNPV |

+: identity obtained by BLASTX.
a: unique gene
*: not annotated in the Genbank database genome
§: acquired by manual alignment using the MAFFT method

**Table S2**. Species used in this paper for reconstruction of the baculovirus phylogeny in the FIG. 2. The species from the genera *Alphabaculovirus* from Group I (red) and Group II (black), *Betabaculovirus* (dark blue), *Gammabaculovirus* (orange), and *Deltabaculovirus* (light blue) are presented here together with abbreviation used in the main text, host family from where the virus was isolated, and the Genbank accession number.

| **Species** | **Abbreviation** | **Host family** | **Accession** |
|---|---|---|---|
| *Adoxophyes honmai nucleopolyhedrovirus* | AdhoNPV | Tortricidae | AP006270 |
| *Adoxophyes orana nucleopolyhedrovirus* | AdorNPV | Tortricidae | EU591746 |
| *Agrotis ipsilon multiple nucleopolyhedrovirus* strain illinois | AgipMNPV | Noctuidae | EU839994 |
| *Agrotis segetum nucleopolyhedrovirus* | AgseNPV | Noctuidae | DQ123841 |
| *Apocheima cinerarium nucleopolyhedrovirus* | ApciNPV | Geometridae | FJ914221 |
| *Buzura suppressaria nucleopolyhedrovirus* | BusuNPV | Geometridae | KF611977 |
| *Chrysodeixis chalcites nucleopolyhedrovirus* | ChchNPV | Noctuidae | AY864330 |
| *Clanis bilineata nucleopolyhedrovirus* | ClbiNPV | Sphingidae | DQ504428 |
| *Ectropis obliqua nucleopolyhedrovirus* strain A1 | EcobNPV-A1 | Geometridae | DQ837165 |
| *Euproctis pseudoconspersa nucleopolyhedrovirus* | EupsNPV | Lymantriidae | FJ227128 |
| *Helicoverpa armigera multiple nucleopolyhedrovirus* | HaMNPV | Noctuidae | EU730893 |
| *Helicoverpa armigera nucleopolyhedrovirus* C1 | HaNPV-C1 | Noctuidae | AF303045 |
| *Helicoverpa zea single nucleopolyhedrovirus* USA | HzSNPV-USA | Noctuidae | AF334030 |
| *Hemileuca sp. nucleopolyhedrovirus* | HespNPV | Saturniidae | KF158713 |
| *Lambdina fiscellaria nucleopolyhedrovirus* | LafiNPV | Geometriidae | KP752043 |
| *Leucania separata nuclear polyhedrovirus* strain AH1 | LeseNPV | Noctuidae | AY394490 |
| *Lymantria díspar multiple nucleopolyhedrovirus* | LdMNPV | Lymantriidae | AF081810 |
| *Lymantria xylina multiple nucleopolyhedrovirus* | LyxyMNPV | Lymantriidae | GQ202541 |
| *Mamestra brassicae multiple nucleopolyhedrovirus* strain Chb1 | MbMNPV-CHb1 | Noctuidae | JX138237 |

| | | | |
|---|---|---|---|
| *Mamestra configurata nucleopolyhedrovirus*-A strain 90/2 | MacoNPV-A 90/2 | Noctuidae | U59461 |
| *Mamestra configurata nucleopolyhedrovirus* B | MacoNPV-B | Noctuidae | AY126275 |
| *Orgyia leucostigma nucleopolyhedrovirus* isolate CFS-77 | OrleNPV | Lymantriidae | EU309041 |
| *Peridroma sp. nucleopolyhedrovirus* | PespNPV | Noctuidae | KM009991 |
| ***Perigonia lusca single nucleopolyhedrovirus*** | **PeluSNPV** | **Sphigidae** | **KM596836** |
| *Pseudoplusia includens single nucleopolyhedrovirus* IE | PsinSNPV | Noctuidae | KJ631622 |
| *Spodoptera exigua nucleopolyhedrovirus* | SeMNPV | Noctuidae | AF169823 |
| *Spodoptera frugiperda multiple nucleopolyhedrovirus* isolate 19 | SfMNPV-19 | Noctuidae | EU258200 |
| *Spodoptera litoralis nucleopolyhedrovirus* isolate AN1956 | SpliNPV-1956 | Noctuidae | JX454574 |
| *Spodoptera litura nucleopolyhedrovirus* G2 | SpliNPV-G2 | Noctuidae | AF325155 |
| *Spodoptera litura nucleopolyhedrovirus* II | SpliNPV-II | Noctuidae | EU780426 |
| *Sucra jujuba nucleopolyhedrovirus* | SujuNPV | Geometridae | KJ676450 |
| *Trichoplusia ni single nucleopolyhedrovirus* | TnSNPV | Noctuidae | DQ017380 |
| *Autographa californica nucleopolyhedrovirus* clone C6 | AcMNPV-C6 | Noctuidae | L22858 |
| *Anticarsia gemmatalis nucleopolyhedrovirus* | AgMNPV | Noctuidae | DQ813662 |
| *Antheraea pernyi nucleopolyhedrovirus* isolate L2 | AnpeNPV-L2 | Saturniidae | EF207986 |
| *Bombyx mori nucleopolyhedrovirus* strain T3 | BmNPV-T3 | Bombycidae | L33180 |
| *Bombyx mandarina nucleopolyhedrovirus* S2 | BomaNPV-S2 | Bombycidae | JQ071499 |
| *Choristoneura fumiferana defective multiple nucleopolyhedrovirus* | CfDEFMNPV | Tortricidae | AY327402 |
| *Choristoneura fumiferana multiple nucleopolyhedrovirus* | CfMNPV | Tortricidae | AF512031 |
| *Choristoneura murinana nucleopolyhedrovirus* | ChmuNPV | Tortricidae | KF894742 |
| *Choristoneura occidentalis nucleopolyhedrovirus* | ChocNPV | Tortricidae | KC961303 |
| *Choristoneura rosaceana nucleopolyhedrovirus* | ChroNPV | Tortricidae | KC961304 |
| *Condylorrhiza vestigialis multiple nucleopolyhedrovirus* | CoveMNPV | Crambidae | KJ631623 |

| | | | |
|---|---|---|---|
| *Dendrolimus kikuchii nucleopolyhedrovirus* | DekiNPV | Lasiocampidae | JX193905 |
| *Epiphyas postvittana nucleopolyhedrovirus* | EppoNPV | Tortricidae | AY043265 |
| *Hyphantria cunea nucleopolyhedrovirus* | HycuNPV | Arctiidae | AP009046 |
| *Maruca vitrata multiple nucleopolyhedrovirus* | MaviMNPV | Crambidae | EF125867 |
| *Orgyia pseudotsugata multiple nucleopolyhedrovirus* | OpMNPV | Lymantriidae | U75930 |
| *Philosamia cynthia ricini nucleopolyhedrovirus* | PhcyNPV | Saturniidae | JX404026 |
| *Plutella xylostella multiple nucleopolyhedrovirus* isolate CL3 | PlxyMNPV | Plutellidae | DQ457003 |
| *Rachiplusia ou multiple nucleopolyhedrovirus* | RoMNPV | Noctuidae | AY145471 |
| *Thysanoplusia orichalcea nucleopolyhedrovirus* | ThorNPV | Noctuidae | JX467702 |
| *Adoxophyes orana granulovirus* | AdorGV | Tortricidae | AF547984 |
| *Agrotis segetum granulovirus*-L1 | AgseGV-L1 | Noctuidae | KC994902 |
| *Choristoneura occidentalis granulovirus* | ChocGV | Tortricidae | DQ333351 |
| *Clostera anastomosis granulovirus* | CaLGV | Notodontidae | KC179784 |
| *Clostera anachoreta granulovirus* | ClanGV | Notodontidae | HQ116624 |
| *Cryptophlebia leucotreta granulovirus* isolate CV3 | CrleGV | Tortricidae | AY229987 |
| *Cydia pomonella granulovirus* | CpGV | Tortricidae | U53466 |
| *Epinotia aporema granulovirus* | EpapGV | Tortricidae | JN408834 |
| *Erinnyis ello granulovirus* | ErelGV | Sphingidae | KJ406702 |
| *Helicoverpa armigera granulovirus* | HaGV | Noctuidae | EU255577 |
| *Phthorimaea operculella granulovirus* | PhopGV | Gelechiidae | AF499596 |
| *Pieris rapae granulovirus* E3 | PiraGV-E3 | Pieridae | GU111736 |
| *Plutella xylostella granulovirus* | PlxyGV | Plutellidae | AF270937 |
| *Pseudaletia unipuncta granulovirus* | PsunGV-Hawaiin | Noctuidae | EU678671 |
| *Spodoptera frugiperda granulovirus* | SpfrGV | Noctuidae | KM371112 |

| | | | |
|---|---|---|---|
| *Spodoptera litura granulovirus* isolate K1 | SpliGV | Noctuidae | DQ288858 |
| *Xestia c-nigrum granulovirus* | XcGV | Noctuidae | AF162221 |
| *Neodiprion sertifer nucleopolyhedrovirus* | NeseNPV | Diprionidae | AY430810 |
| *Neodiprion lecontei nucleopolyhedrovirus* | NeleNPV | Diprionidae | AY349019 |
| *Neodiprion abietis nucleopolyhedrovirus* | NeabNPV | Diprionidae | DQ317692 |
| *Culex nigripalpus nucleopolyhedrovirus* | CuniNPV | Culicidae | AF403738 |

**Table S3**. Category of the AcMNPV homologs found in the PeluSNPV genome.

| **Category** | **Genes** |
|---|---|
| Structural | *Polyhedrin* (*polh*, *pelu001*, occlusion body protein), *orf1629* (*pelu002*, nuclear actin assembly), *pk-1* (*pelu003*), *occlusion derived virus envelope protein 18* (*odv-e18*, *pelu013*), *occlusion derived virus enveloped protein 27* (*odv-e27*, *pelu014*), *p10* (*pelu030*, fibrous protein related to OB formation), *few polyhedra protein/25k* (*fp25k*, *pelu041*), *viral protein 1054* (*vp1054*, *pelu049*, capsid protein), *desmoplakin* (*pelu068*), *gp41* (*pelu074*, tegument protein), *vp91/p95* (*pelu077*), *vp39* (*pelu079*, major capsid protein), *p33* (*pelu081*, sulfhydryl oxidase), *odv-e25* (*pelu083*), *p87/vp80* (*pelu093*, capsid), *odv-ec43*(*pelu095*), *p13* (*pelu097*), *odv-e66* (*pelu134*), *calyx/polyhedrin enveloped protein* (*calyx/pep*, *pelu125*), *p24* (*pelu121*), *per os infectivity factor 0* (*p74/pif-0*, *pelu008*), *pif-1* (*pelu133*), *pif-2* (*pelu115*), *pif-3* (*pelu107*), *odv-e28/pif-4* (*pelu085*), *odv-e56/pif-5* (*pelu020*), *f protein* (*pelu135*) |
| Replication | **Essential**: *dna-polymerase* (*pelu069*), *late expression factor 1* (*lef-1*, the virus primase, *pelu142*), *lef-2* (*pelu117*, the primase accessory fator), *lef-3* (*pelu067*, a single-stranded DNA binding protein), *lef-11* (*pelu024*), *p6.9* (*pelu088*, arginine, serine, threonine-rich DNA binding protein for DNA packaging),*38 k* (*pelu086*), *dna-helicase* (*pelu084*), and *immediate early gene 1* (*ie-1*, *pelu019*). **Non-essential**: *major early-transcribed protein 53* (*me53*, *pelu009*), *DNA binding protein 1* (*dbp-1*, *pelu022*), *alkaline exonuclease* (*alk-exo*, *pelu129*) and *exon-0/ie-0* (*pelu011*). |
| Transcription | *lef-4* (*pelu080*, the RNA capping protein), *lef-8* (*pelu057*, an RNA polymerase subunit), *lef-9* (*pelu040*, an RNA polymerase subunit), and *p47* (*pelu021*, an RNA polymerase subunit), *lef-5* (*pelu087*, TFIIS related protein), *very late factor 1* (*vlf-1*) (*pelu072*), *lef-6* (*pelu033*, presents an RNA binding domain of the mRNA export factor TAP), *39K* (*pelu025*), *lef-10* (*pelu050*) and *protein kinase 1* (*pk-1*, *pelu003*) |
| Auxiliary | *hoar* (*pelu005*, with a RING-finger domain), *chtb-1* (*pelu015*), *v-ubiquitin* (*pelu027*), *p26-1* (*pelu031*), *dbp-2* (*pelu034*), *conotoxin-like 1* (*ctl-1*, *pelu039*), *baculovirus repeated ORF A* (*bro-a*, *pelu042*), *chab-a* (*pelu043*), *chab-b* (*pelu044*), *gp37* (*pelu058*), *chitinase* (*pelu060*), *v-cathepsin* (*pelu061*), *p26-2* (*pelu062*), *chtb-2* (*pelu063*), *inhibitor of apoptosis 2* (*iap-2*, *pelu064*), *methyltransferase 1* (pelu065), *iap-3* (*pelu102*), *glycohydrolase* (*parg*, *pelu105*), *ctl-2* (*pelu111*), *tmk-dut* (*pelu112*), *he65* (*pelu124*), *rr2a* (*pelu126*), *ecdysteroid UDP-glucosyltransferase* (*egt*, (*pelu139*), *rr1* (*pelu145*) |
